# Telomere damage-mediated senescence in alveolar epithelial type II cells but not in macrophages aggravates inflammation in acute lung injury

**DOI:** 10.1186/s12931-026-03627-0

**Published:** 2026-03-14

**Authors:** Christina Beatrix Hildebrand, Seyma Öztürk, Jia Li Ye, Dirk Wedekind, Ulrich A. Maus, Christian Bär, Christian Mühlfeld, Christina Brandenberger

**Affiliations:** 1 Institute of Functional Anatomy, Charité-Universitätsmedizin Berlin, Philippstraße 11, 10115 Berlin, Germany; 2 Institute of Functional and Applied Anatomy, Hannover Medical School, Carl-Neuberg-Strasse 1, Hannover, 30625 Germany; 3 German Center for Lung Research (DZL), partner site Berlin, Berlin, Germany; 4 Institute of Molecular and Translational Therapeutic Strategies, Hannover Medical School, Carl-Neuberg-Strasse 1, Hannover, 30625 Germany; 5 Institute of Laboratory Animal Science, Hannover Medical School, Carl-Neuberg-Strasse 1, Hannover, 30625 Germany; 6 Department of Experimental Pneumology, Hannover Medical School, Feodor- Lynen-Strasse 21, Hannover, 30625 Germany; 7 Biomedical Research inEndstage and Obstructive Lung Disease Hannover (BREATH), Member of the German Center for Lung Research (DZL), Hannover, Germany; 8 Fraunhofer Institute for Toxicology and Experimental Medicine (ITEM), Nikolai-Fuchs-Str. 1, Hannover, 30625 Germany

**Keywords:** acute lung injury, pulmonary aging, cellular senescence, alveolar epithelial type II cells, macrophages, telomere distortion, inflamm-aging

## Abstract

Severity of acute lung injury (ALI) increases with age and is associated with enhanced inflammation in older individuals (inflamm-aging), characterized by increased expression of pro-inflammatory cytokines. Telomere damage can cause irreversible cell cycle arrest, known as cellular senescence, that may promote pro-inflammatory signaling and exacerbate ALI. Therefore, we investigated the role of telomere damage-mediated senescence in alveolar epithelial type II (AEII) cells and macrophages in injury and inflammation of lipopolysaccharide (LPS)-induced ALI in mice.

Cell type-specific deletion of *Trf1* (telomeric repeat-binding factor 1) was achieved using tamoxifen-inducible Cre-loxP mouse models driven by the Sftpc (surfactant protein c) promoter for AEII cells or Lyz2 (lysozyme 2) promoter for macrophages to induce telomere damage in respective cell types. Mice without Trf1 deletion were used for comparison, as well as naturally aged mice. After tamoxifen injections, ALI was induced by intranasal application of 2.5 mg LPS/kg body weight. Control mice received saline. The severity of ALI, as well as cell type-specific molecular responses, were assessed using functional, immunological, histological and transcriptomic investigations.

Telomere damage-mediated senescence in AEII cells resulted in an increase in inflammatory BALF cells and cytokines and a decline in lung function 72 h after LPS exposure, compared to mice without Trf1 deletion in AEII cells. Transcriptomic analysis revealed that Trf1-deleted AEII cells exhibited specific cell cycle inhibition, as well as an elevated DNA damage response (DDR), in both control and LPS-treated Sftpc-Cre mice. Trf1 deletion in macrophages triggered an augmented DDR without affecting LPS-driven lung inflammation compared to mice without Trf1 deletion. Control mice with Trf1 deletion of both Cre-loxP mouse models did not show signs of inflamm-aging. Additional comparison with old mice revealed that global aging causes a much greater severity in ALI than Trf1 deletion in lung cells.

The findings demonstrate that telomere damage-mediated senescence in AEII cells promotes inflammation in the late exudative phase of ALI, while Trf1 deletion in macrophages did not cause an aggravation of ALI. The results also suggest that additional mechanisms of aging beyond pulmonary telomere damage and cellular senescence, such as global inflamm-aging, contribute to the severity of ALI in old age.

**Supplementary Information:**

The online version contains supplementary material available at 10.1186/s12931-026-03627-0.

## Background

Acute respiratory distress syndrome (ARDS) is a severe pulmonary condition characterized by a rapid onset of pulmonary inflammation and impaired gas exchange with respiratory failure and death [1]. ARDS is frequently caused by severe sepsis or pneumonia, while also including severe conditions of Covid-19 [2, 3]. It can affect individuals of all ages, but especially patients over 65 years of age are more likely to develop ARDS with a poor survival rate [4, 5]. Also, in animal models of acute lung injury (ALI) that mimic main features of ARDS such as exacerbation of inflammation, injury of the air-blood barrier, edema formation and impaired lung function, an increased severity of injury and higher mortality rate was observed in older mice [6–8].

Aging is associated with an accumulation of senescent cells, which are characterized by an irreversible cell cycle arrest and profound alterations in chromatin organization and gene expression [9]. Senescent cells are functionally impaired, but still viable and metabolically active [9] and have been described to secrete a series of pro-inflammatory cytokines, chemokines, growth factors, proteases, and matrix remodeling enzymes - collectively termed as the senescence associated secretory phenotype (SASP) [10, 11]. SASP in turn is suggested to contribute to a chronic low-grade inflammation in the elderly – also called inflamm-aging [12], which is associated with an increased severity of developing ARDS/ALI [4, 13]. Senescence-related functional impairment of different cell populations in the lung might further enhance the severity of the disease and treatments with senolytics/senomorphics could represent potential approaches to target age-related severity in ARDS/ALI.

Pulmonary macrophages and alveolar epithelial type II (AEII) cells play a crucial role in the development and regeneration of ALI. Macrophages are responsible for clearing invading pathogens and initiating the inflammatory response by releasing inflammatory chemokines to recruit other immune cells [14, 15]. In addition, they phagocytose cell debris and apoptotic cells and contribute to the termination of inflammation and repair after injury [16]. Evidence suggests that alveolar macrophage function is impaired in old mice [17, 18], and a shift towards the pro-inflammatory M1 phenotype has been observed [19, 20]. These age-related changes in alveolar macrophages (AMs) could therefore contribute to an enhanced inflammatory signaling in ALI with increased age. On the other hand, AEII cells serve as progenitor cells of alveolar epithelial type I (AEI) cells, which are essential for restoring the integrity and functionality of the blood-air barrier after lung injury [21]. Moreover, they synthesize and release pulmonary surfactant, which reduces surface tension at the air-liquid interphase and prevents alveolar collapse [22]. Previous studies have shown an impaired AEII cell function and senescence with aging - in particular in chronic (age-related) lung diseases such as COPD and pulmonary fibrosis [7, 11], and that senescence in AEII cells contributes to delayed regeneration after lung injury [23]. However, whether or not cellular senescence in AEII cells or macrophages promotes acute inflammation in ARDS/ALI is still poorly defined. In the current study, we therefore examined the effect of telomere damage-mediated cellular senescence in macrophages and AEII cells on the inflammatory response in the lung to endotoxin.

The induction of telomere damage-mediated senescence was achieved by cell type-specific knockout of TRF1 (telomeric repeat-binding factor 1), a protein within the shelterin complex, which protects telomeres [24, 25]. Knockout of *Trf1* leads to chromosomal instability and cellular senescence [26, 27], as shown in previous studies where *Trf1* knockout in AEII cells induced markers of cellular senescence such as *Cdkn1a* (cyclin dependent kinase inhibitor 1 A; p21) and *Trp53* (tumor protein 53; p53), limited the cellular proliferative capacity and promoted the development of pulmonary fibrosis in mice [23, 26, 28].

In this study, the hypothesis was tested that senescence in AEII cells or macrophages, mediated by cell type-specific telomere distortion, promotes inflamm-aging and aggravates lung injury in the acute phase of injury that is particularly critical for immediate survival in old mice. Our findings demonstrate that Trf1 deletion in AEII cells increases the inflammatory response during the late exudative phase of ALI, whereas Trf1 deletion in macrophages does not exacerbate ALI. Comparisons with naturally aged mice suggest that further mechanisms of aging beyond pulmonary telomere-damage mediated cellular senescence, such as global inflamm-aging, contribute to the severity of ALI in old age.

## Materials and methods

### Mouse strains and experimental design

Prior to generation of the Cre-LoxP mouse lines, individual transgenic mouse lines (#028054; #032291; #007909; #012336) were purchased from Jackson Laboratory (Bar Harbor, ME) and standardized to the C57BL/6J background. For induction of telomere damage-mediated cellular senescence in AEII cells, Trf1 deletion was performed by Cre-Recombinase expression under the Sftpc (surfactant protein c) promoter and for macrophages under the Lyz2 (lysozyme 2) promoter. Of note, the later also targets other cells of myeloid lineages such as monocytes or neutrophils. In addition, a strain with Cre-inducible expression of tdTomato, a red fluorescent protein (Ai9), was bred in for tracing Cre-expressing cells. Four different mouse strains were used for the experiments: Sftpc-Ai9, Sftpc-Ai9-Trf1, Lyz2-Ai9 and Lyz2-Ai9-Trf1 mice. The mice were mated and bred as shown in the supplemental information (strain details and figure S1) and as described previously [23]. The model was established at the Institute of Functional and Applied Anatomy of Hannover Medical School, and mice were bred in the local housing facility. All animal procedures were approved by the Lower Saxony State Office for Consumer Protection and Food Safety, in accordance with the German Animal Welfare Act and the European Directive 2010/63/EU.

Cre-recombinase expression was induced by tamoxifen treatment. Due to possible side effects by tamoxifen injection in female mice, only male mice were used in the experiments at an age of 3 months, unless noted otherwise. At experimental start, all mice received intraperitoneal tamoxifen (#T5648, Sigma Aldrich) injections on days 1 and 3 at a dosage of 3 mg tamoxifen/150 µl corn oil (#C8267, Sigma Aldrich). One week after the second tamoxifen injection, ALI was induced by intranasal application of 2.5 µg lipopolysaccharide (LPS, O111:B4, Sigma-Aldrich)/g body weight. Control animals received saline only. After 24–72 h, the mice were euthanized.

### Lung mechanics measurement and lung tissue harvest

Prior to killing, mice were anesthetized and lung function measurements were conducted with a FlexiVent to determine the static compliance (Cst), tissue resistance (G) and elastance (H) as described previously [8]. Afterwards, bronchoalveolar lavage fluid (BALF) was collected for cytokine and cytometry analysis, and lung tissue was harvested for histopathology or gene expression analysis to determine the severity of the induced lung injury. The cranial and middle lung lobe were snap frozen in liquid nitrogen and stored at -80 °C till further analysis. For histological processing, the left, caudal and accessory lung lobes were instilled with 4% paraformaldehyde (PFA) solved in phosphate buffer at a pressure of 20 cm H2O and stored in the fixative at 4 °C over-night. After 24 h, the caudal and accessory lobes were washed and embedded in OCT for cryo-sections and immunofluorescence (IF) as described previously [23]. The left lobe was post-fixed with 1.5% PFA and 1.5% glutaraldehyde in 0.15 mM HEPES buffer, cut into 2 mm sections and embedded in glycol methacrylate (Technovit 7100 resin, Kulzer GmbH) for light microscopic analysis and histopathology as described previously [8].

### BALF cytometry and cytokine analysis

For BALF collection, lungs were flushed twice with 0.7 ml saline solution. The BALF was centrifuged (4 °C, 400 g, 15 min) and the resulting supernatant was aliquoted and stored at -80 °C. Lysis of erythrocytes in the collected BALF cells was done with the RBC-Lysis-Buffer (#420301, BioLegend) according to the supplier’s manual. Afterwards, cells were suspended in 1 ml PBS. The total number of BALF cells was counted using a Neubauer chamber and cell differential analysis to quantify BALF macrophages and neutrophils was done by generating and analyzing Cytospins stained with the DiffQuick staining kit (Medion Diagnostics) as described previously [19]. Cytokines in the BALF supernatant were quantified by using a 13-plex cytokine bead array (LEGENDplex™, BioLegend) according to the manufacturer`s instructions. The following cytokines were analyzed: IL-6 (interleukin 6), CCL2 (CC-chemokin-ligand 2), CXCL1 (C-X-C motif chemokine ligand 1), GM-CSF (granulocyte-macrophage colony-stimulating factor), and TNFα (tumor necrosis factor alpha). Note that cytokine analysis was only done in LPS-treated animals, as cytokine levels in BALF of control mice are usually below the assay detection limit [19].

### Real-time PCR

Gene expression analysis was performed on the snap frozen middle lung lobe. RNA isolation was carried out utilizing the NucleoSpin™ RNA Mini Kit (Machery Nagel) according to the manufacturer’s instructions. The concentration of RNA was determined using the NANO-DROP 2000 spectrophotometer (Thermo Scientific). Subsequently, 1 µg isolated RNA was transcribed into cDNA using the iScript™ cDNA Synthesis Kit (Bio-Rad) following the manufacturer’s instructions. Real-time PCR was performed with a C100 Thermal Cycler (CFX96 Real-Time System, Bio-Rad) and a thermocycling protocol consisting of 40 cycles of 95 °C for 5 s and 60 °C for 20 s. The used primer sequences are displayed in Table 1. The relative mRNA expression of genes of interest was assessed by normalization to the housekeeping gene *Hprt1* (hypoxanthin-phosphoribosyl-transferase 1) [7].

**Table 1:** List of used primers

**Gene**	**Primer/ Assay ID**	**Company**
*Hprt *(forward)	CCTAAGATGAGCGCAAGTTGAA	Eurofins Genomics
*Hprt *(reverse)	CCACAGGACTAGAACACCTGCTAA	
*Cdkn2a *(p16)	qMmuCED0038108	Biorad
*Cdkn1a *(p21)	qMmuCEP0054691	
*Trp53 *(p53)	qMmuCIP0032520	
*Trf1*	qMmuCEP0055044	

### Single-cell RNA sequencing analysis

Single-cell RNA sequencing (scRNAseq) was performed according to a protocol based on Yazicioglu et al. and adapted to our experimental setup [7]. Lungs were harvested from both Sftpc-Cre and Lyz2-Cre mice with and without Trf1 deletion that were treated with either saline or LPS (24 h exposure). Lungs were digested and a single cell suspension was generated as described before [7]. In brief, the lungs were instilled with 1.5 ml of dispase (5000U, Corning #3544235) and 200 µl low-melting agarose, excised and further incubated in dispase for 45 min. Airways were removed and the lungs were minced and incubated with 0.5% DNAse for 10 min. Afterwards, cell suspension was prepared and the collected cells were filtered and washed. In a next step, the cells were incubated on ice for 15 min with an anti-mouse CD16/32 blocking antibody (1:50, #101301, BioLegend), followed by a 30-minute incubation with a pre-incubated mixture of biotinylated anti-mouse CD326 (1:200, #118203, BioLegend), biotinylated anti-mouse CD64 (1:100, #139318, BioLegend) and oligo-barcoded PE streptavidin TotalSeq-A antibodies (1:200, BioLegend) that has been prepared in accordance with the supplier’s manual. For each experimental group, a different HashTag oligo-barcoded antibody was used: Ai9 control (#405251), Ai9-Trf1 control (#405253), Ai9 LPS (#405255), Ai9-Trf1 LPS (#405257). In a next step, cells were washed and incubated for another 30 min with an anti-PE APC antibody (1:100, #408108 BioLegend) and 5 min with DAPI. The cells were then sorted for APC positive and DAPI negative populations, counted, and the four experimental groups were pooled in equal number for scRNAseq analysis and loaded onto a 10x Genomics lane. Library generation, sequencing run and raw data processing were furthermore done as described [7]. The analysis and visualization were conducted with 10x Genomics Loupe Browser 6.5.0. Cell type identification across clusters was achieved by referencing established marker genes, as reported in the single-cell transcriptomic atlas of the aging mouse lung [29]. Specifically, AEII cells, AMs and monocytes/interstitial macrophages (Mo/IMs) were clustered and assigned to the four experimental groups, separated based on the HashTag-derived Oligos and by using the filter tool. A comparative feature analysis was then performed within these three cell populations and experimental groups to identify genes with significantly changed expression in each cluster relative to the others. The gene expression tool was used to examine the expression profiles of genes involved in DNA damage response (DDR) and inflamm-aging. Heat-maps were generated showing the relative gene expression in each cluster. Genes with very low cellular abundance in all clusters were excluded.

### Confirmation of cell specific Trf1 deletion

To confirm specificity of Trf1 deletion in tdTomato positive cells of Sftpc-Ai9-Trf1 and Lyz2-Ai9-Trf1 mice, lungs were digested to a single cell suspension and sorted as described previously [23]. Afterwards, RNA was isolated from these cells with the ReliaPrep™ miRNA Cell and Tissue Miniprep System (Z6212, Promega) isolation kit according to the manufacturer’s instructions. Afterwards, transcription into cDNA was performed using the iScript™ cDNA Synthesis Kit (#1708891, Bio-Rad). The expression of *Trf1* was compared between mice with and without knockout.

### Immunofluorescence for cell specific Cre-expression analysis

For IF staining, OCT embedded lung tissue was sectioned into 5 μm thick slices. Sections were stained with tdTomato as described in our previous study [23]. Slides were incubated overnight at 4 °C with anti-pro-SP-C (rabbit, WRAB-76694, Seven Hills) to detect AEII cells or CD68 (rabbit, ab125212, Abcam) to detect macrophages at a dilution of 1:500 in a goat serum mixture (1% BSA/PBS, 5% goat serum, 0.3% Triton X-100). The next day, the slides were incubated with Alexa 488 goat anti-rabbit (A-11008, Invitrogen) at a dilution of 1:1000 in 1% BSA/PBS and 0.3% Triton X-100 for 60 min and stained with DAPI (1:1000) for 10 min prior to mounting in Mowiol. The slides were scanned with an AxioScan.Z1scanner (Zeiss) and analyzed using systematic uniform random sampling at 40x magnification.

### Immunofluorescence staining for p21 staining

The presence of p21-expressing tdTomato positive AEII cells and macrophages in lung tissue was determined on OCT embedded cryo-sections, using IF as described in Hirsch et al. [23]. Tissue slides were incubated overnight with rabbit anti-p21 antibody (ab188224, Abcam) at a 1:500 dilution in a goat serum mixture (1% BSA/PBS, 5% goat serum, 0.3% Triton X-100). The following day, the secondary antibody Alexa 546 goat anti-rabbit (A-11035, Invitrogen) was incubated for 60 min at a 1:1000 dilution in 1% BSA/PBS and 0.3% Triton X-100 together. Before slides were mounted in Mowiol, tissue sections were incubated with DAPI at a 1:1000 concentration for 5 min. Slides were scanned with an AxioScan.Z1 scanner (Zeiss) at 20x magnification. Quantification was performed starting at a randomly selected location within the section and continued in a meander like pattern (systematic uniform random sampling). At least 52 fields of view, each with a minimum of 100 tdTomato positive cells in Sftpc-Cre mice or 60 in Lyz2-Cre mice, were counted per animal with a 200% digital zoom. The percentage of tdTomato positive cells with a positive p21 signal was then assessed.

### Immunofluorescence staining for γ-H2AX expression

IF staining for histone γ-H2AX was performed on OCT embedded tissue sections that were cut into 4 μm slices and mounted on glass slides. For antigen retrieval, slides were immersed in a 1x Dako Retrieval Solution at pH 6.0 (S1699, Dako) and microwaved at 350–700 W for 7 min, repeated twice. Following retrieval, slides were cooled on ice for 30 min, rinsed with distilled water, and washed with 0.1% Tween-20 in PBS for 5 min. Blocking was performed using a blocking solution containing 5% donkey serum, 1% BSA, and 0.3% Triton X-100 in PBS for 60 min at room temperature. Slides were then washed with 0.1% Tween-20 in PBS twice: first for 5 min, then for 10 min. Primary antibody incubation was performed with goat anti-tdTomato antibody (orb182397, Biorbyt) 1:250 diluted in the blocking solution for 60 min. After washing, slides were incubated with the secondary antibody Alexa Fluor 594 donkey anti-goat (A11058, Invitrogen) 1:500 diluted in blocking solution for 60 min. Subsequently, slides were incubated over-night at 4 °C with anti-γ-H2AX (rabbit, ab11174, Abcam) 1:500 diluted in a solution containing 5% goat serum, 1% BSA, and 0.3% Triton X-100 in PBS. After washing, Alexa Fluor 488 (donkey anti-rabbit, A21206, Invitrogen) 1:500 diluted in blocking solution was applied for 60 min. Slides were washed again with PBS, followed by incubation with Hoechst (H3570, Life Technologies) 1:1000 diluted in blocking solution for 10 min. After additional PBS washes, slides were mounted using ProLongTM Gold antifade reagent (P36930, Life Technologies) mounting medium and imaged. Slides were imaged using a Zeiss LSM 980 confocal microscope with a 40x oil immersion objective. Z-stack were acquired with 0.6 μm thickness to capture the entire nuclear volume. Imaging parameters (laser power, gain, pinhole size) were standardized across samples. At least 50 tdTomoato positive cells were acquired and analyzed per sample using Image J. Data were reported as percentage of double positive cells (γ-H2AX and tdTomato) per total amount of analyzed tdTomato positive cells.

### Statistical analysis

For data analysis the SigmaPlot software (SYSTAT Software, Germany) was used. Group size was *n* = 8–10 per experimental group for saline and *n* = 10–14 per experimental group for LPS exposed animals. Grubbs’ outlier test was performed and maximally one significant outlier per group was excluded. All data were tested for normal distribution using Shapiro-Wilk test. In case of non-normality, a logarithmic- or square root-transformation was applied. A two-way ANOVA or a one-way ANOVA followed by Bonferroni´s post-hoc-test was used to compare strain- and age-effects. *P* ≤ 0.05 was considered statistically significant. For analysis of scRNAseq data, the 10x Genomics Loupe Browser 6.5.0 was used that assigned significance with calculated p-values of *p* ≤ 0.1.

## Results

### Characterization of Trf1 knockout mouse models

**Fig. 1:**
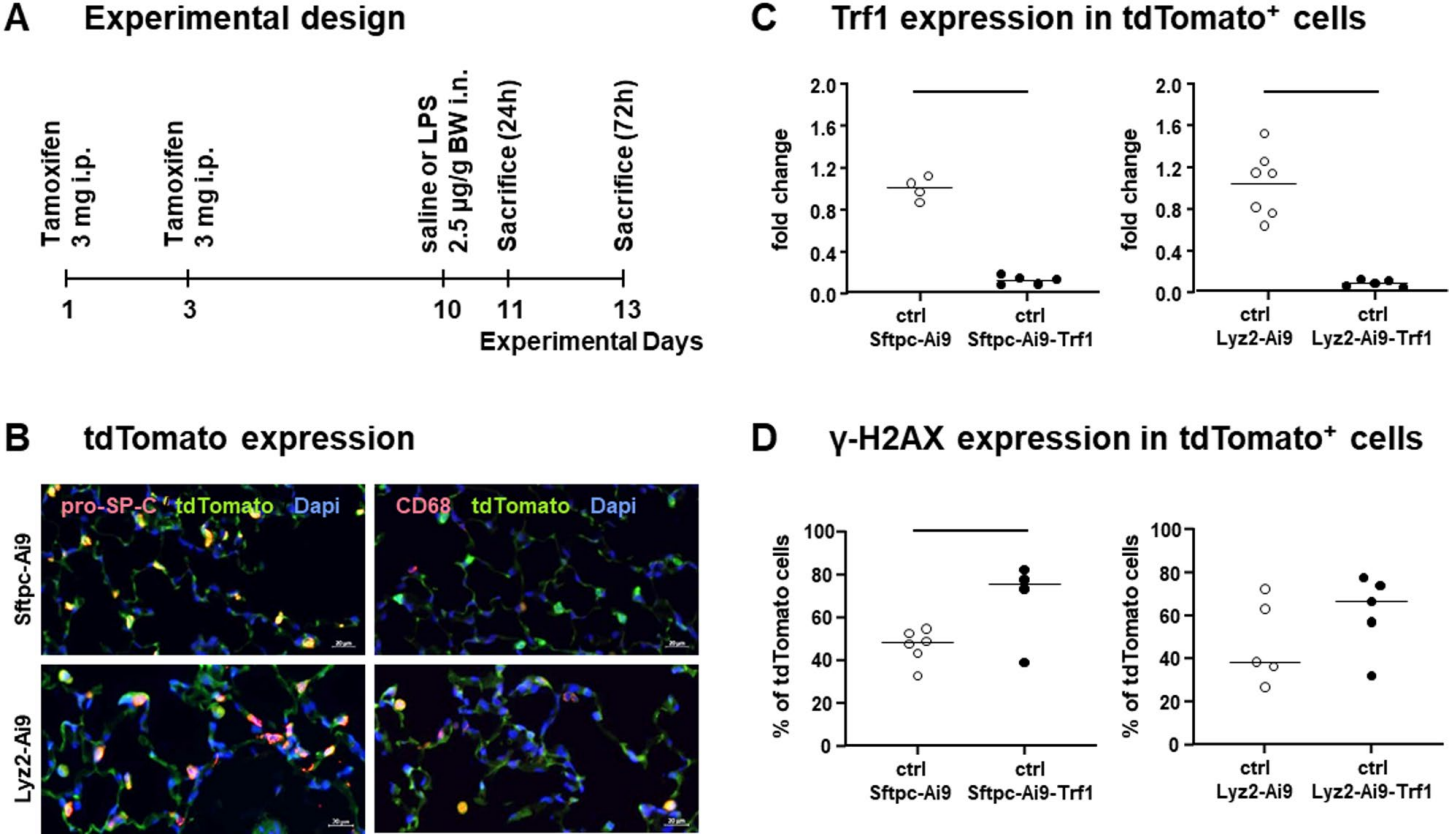
Experimental design and characterization of Trf1 knockout mouse model. Experiments were conducted as outlined in the experimental design (**A**). Promoter specificity was tested by IF to detect tdTomato expression in lung tissue (**B**). AEII cells were detected by pro-SP-C staining (red) and macrophages by CD68 staining (red). TdTomato expression is shown in green and cell nuclei in blue. Trf1 deletion was tested by gene expression analysis in tdTomato positive cells (**C**) and by IF to determine γ-H2AX expression in control Sftpc-Cre and Lyz2-Cre mice (**D**). One-way ANOVA with Bonferroni’s post hoc test was used to compare strain effects with Trf1 deletion. Each data point represents one animal and the line indicates statistically significant difference (*p* ≤ 0.05), *n* = 4–8 mice per group

Trf1 deletion was induced by tamoxifen injections prior to induction of ALI and the experimental setup is displayed in Fig. 1A. The specificity of Sftpc-Cre and Lyz2-Cre promoters was investigated by IF analysis via the expression of tdTomato in AEII cells and macrophages in lung tissue of control mice (Fig. 1B) and the deletion of Trf1 in AEII cells and macrophages by gene expression in respective sorted cell populations (Fig. 1C). IF results showed tdTomato expression in all pro-SP-C positive AEII cells confirming a specific Cre-Recombinase expression under the Sftpc promoter (Fig. 1B). Cre-Recombinase activation related to the Lyz2 promoter identified approximately 70% of all macrophages to be double positive for CD68 and tdTomato. In addition, approximately 30% of AEII cells showed Lyz2 mediated Cre-recombinase activity and tdTomato expression. The Trf1 expression was 8-12-fold reduced in Sftpc-Ai9-Trf1 and Lyz2-Ai9-Trf1 (Fig. 1C). This is in accordance with our previous analysis in Sftpc-Ai9-Trf1 mice, where Trf1 deletion was confirmed by real-time PCR and IF [23]. An additional analysis of DNA damage response with γ-H2AX expression due to Trf1 deletion in Sftpc-Cre and Lyz2-Cre control mice (Fig. 1D) showed γ-H2AX expression was significantly elevated in AEII cells of Sftpc-Ai9-Trf1 mice. In line with the findings reported by Povedano et al. [26, 28, 30], we confirmed Trf1 knockout and observed higher levels of y-H2AX expression in AEII cells and macrophages, although only statistically significant for AEII cells and not for macrophages.

### Impact of Trf1 deletion in AEII cells and macrophages on the development of ALI

**Fig. 2:**
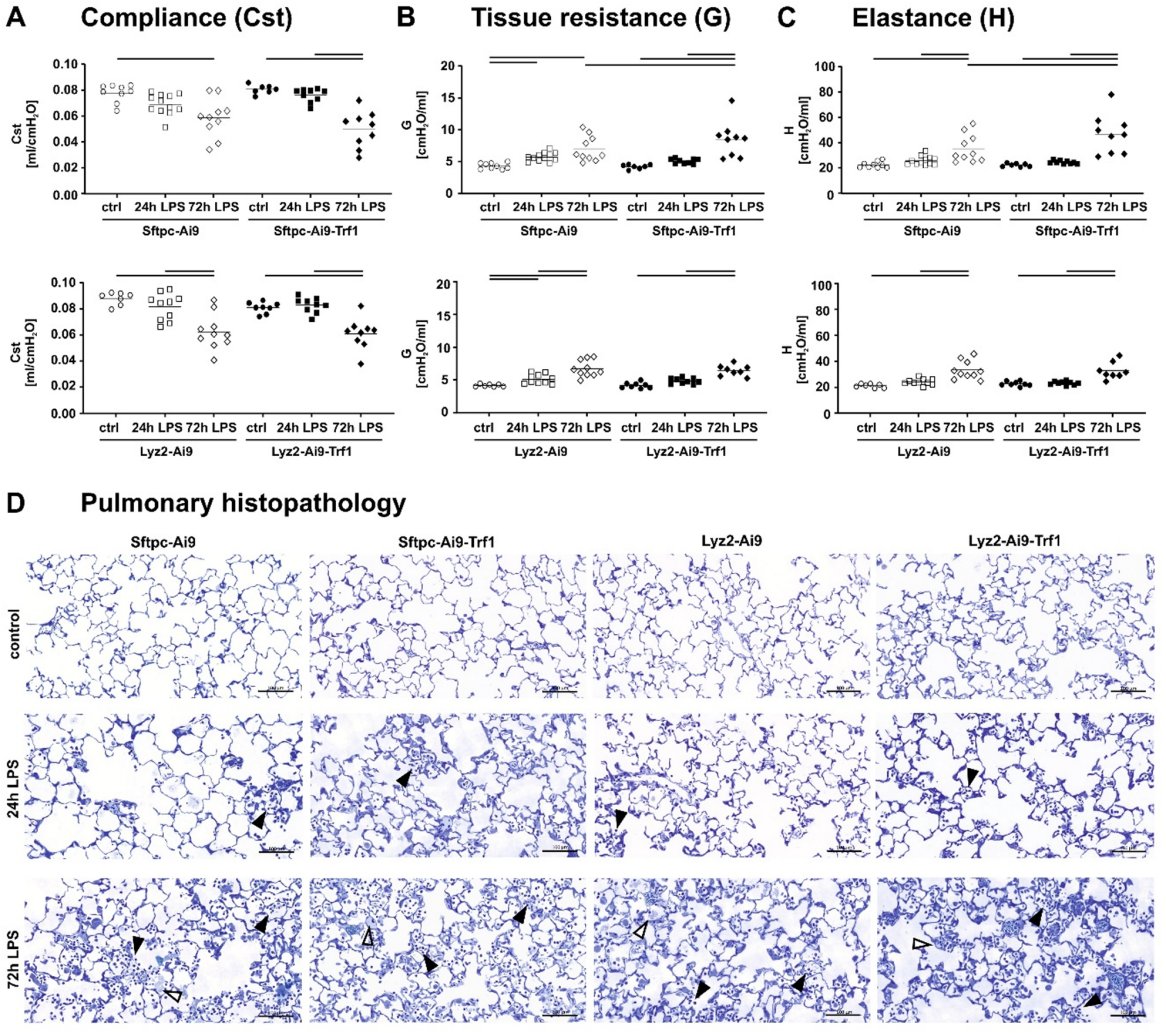
Lung mechanics and histopathology. Lung mechanics, including compliance (**A**), tissue resistance (**B**), and elastance (**C**), were measured in Sftpc-Cre and Lyz2-Cre mice using a FlexiVent ventilator for mice. Two-way ANOVA with Bonferroni’s post hoc test was applied to compare exposure and strain effects. Lines indicate significant differences between experimental groups (*p* < 0.05); *n* = 8–10 for saline and *n* = 11–14 for LPS groups (each data point represents one animal). Representative toluidine blue-stained lung sections of Sftpc-Cre and Lyz2-Cre mice were treated with saline or LPS 24–72 h after exposure (**D**). The arrows indicate infiltrates of immune cells (black arrow) and edema formation (white arrows). Scale bars = 100 μm

The severity of ALI was assessed by measurements of lung mechanics to evaluate the resulting functional consequences of injury. Induction of ALI led to significant alterations in lung mechanics, specifically a decreased compliance (Cst, Fig. 2A) and increased tissue resistance (G, Fig. 2B) and elastance (H, Fig. 2C) in all mouse strains 72 h after LPS exposure. These findings suggest pulmonary inflammation with edema formation, as previously reported [8]. In Sftpc-Ai9-Trf1 mice, a higher increase in G and H was measured 72 h after LPS exposure compared to Sftpc-Ai9 mice (Fig. 2B and C). No significant effects were observed in Lyz2-Ai9-Trf1 mice compared to respective treatment groups without Trf1 deletion. This indicates that Trf1 deletion in AEII cells reduces lung function in ALI over time, while Trf1 deletion in macrophages had no further impact on lung mechanics.

Furthermore, histological imaging of the lung tissue was performed (Fig. 2D), to qualitatively assess the structural alterations in the lung. LPS treatment triggered cellular infiltration in the lung tissue, particularly with neutrophils, which accumulated mainly in the alveoli and in the septal interstitium and protein-rich fluid was observed in the lung parenchyma 72 h after LPS exposure, as an indication of alveolar edema. However, no obvious histopathological differences could be observed due to Trf1 deletion in Sftpc-Cre or Lyz2-Cre mice (Fig. 2D).

### Impact of Trf1 deletion in AEII cells and macrophages on the inflammation in ALI

**Fig. 3:**
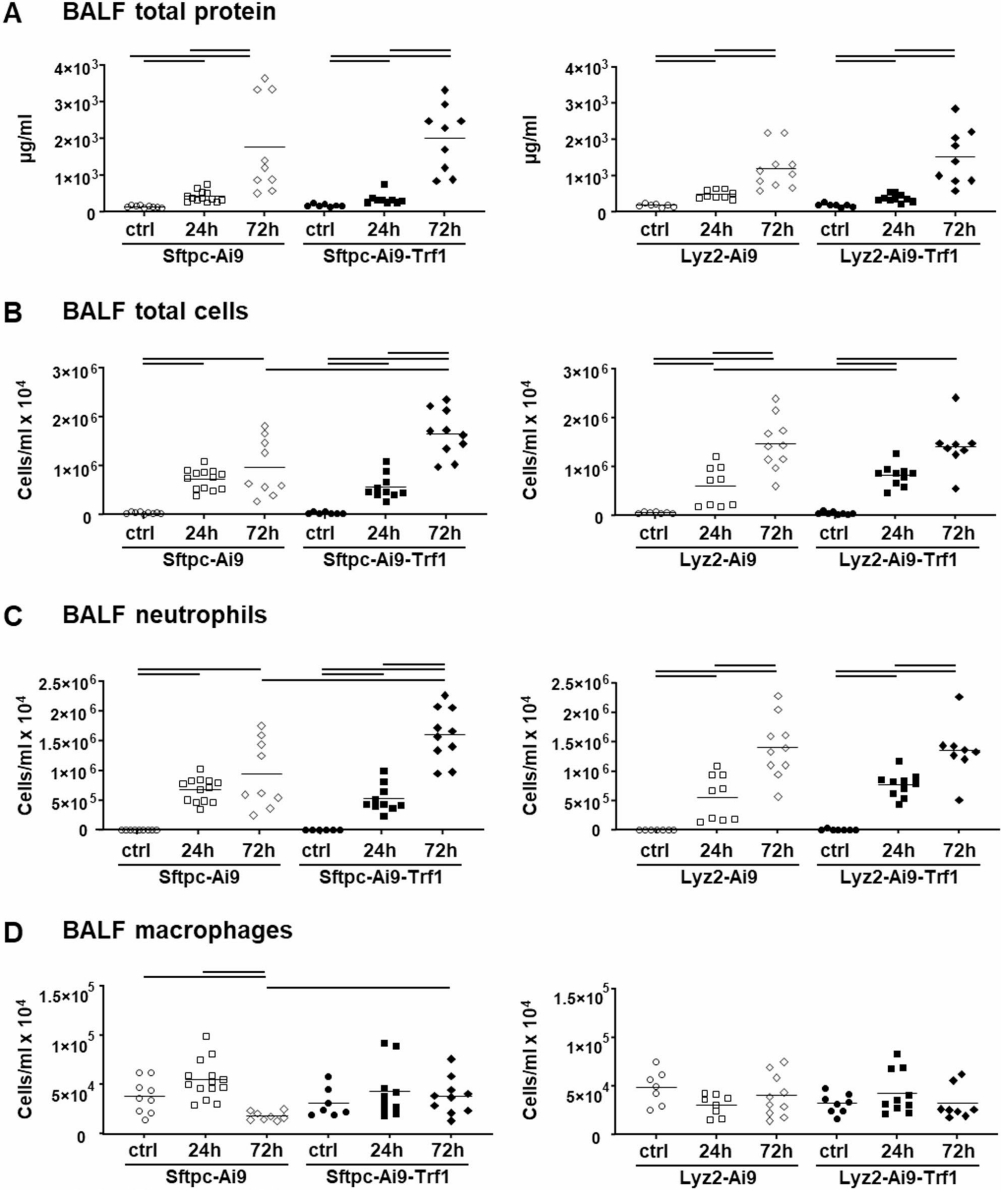
Protein Levels and cytometry in bronchoalveolar lavage fluid (BALF). BALF total protein concentrations (**A**) and differential cell counts of total cells (**B**), neutrophils (**C**) and macrophages (**D**) were determined in Sftpc-Cre and Lyz2-Cre mice. Two-way ANOVA with Bonferroni’s post hoc test was applied to compare exposure and strain effects. Lines indicate significant differences between experimental groups (*p* < 0.05); *n* = 8–10 mice per saline groups and *n* = 11–14 mice per LPS groups (each data point represents one animal)

**Fig. 4:**
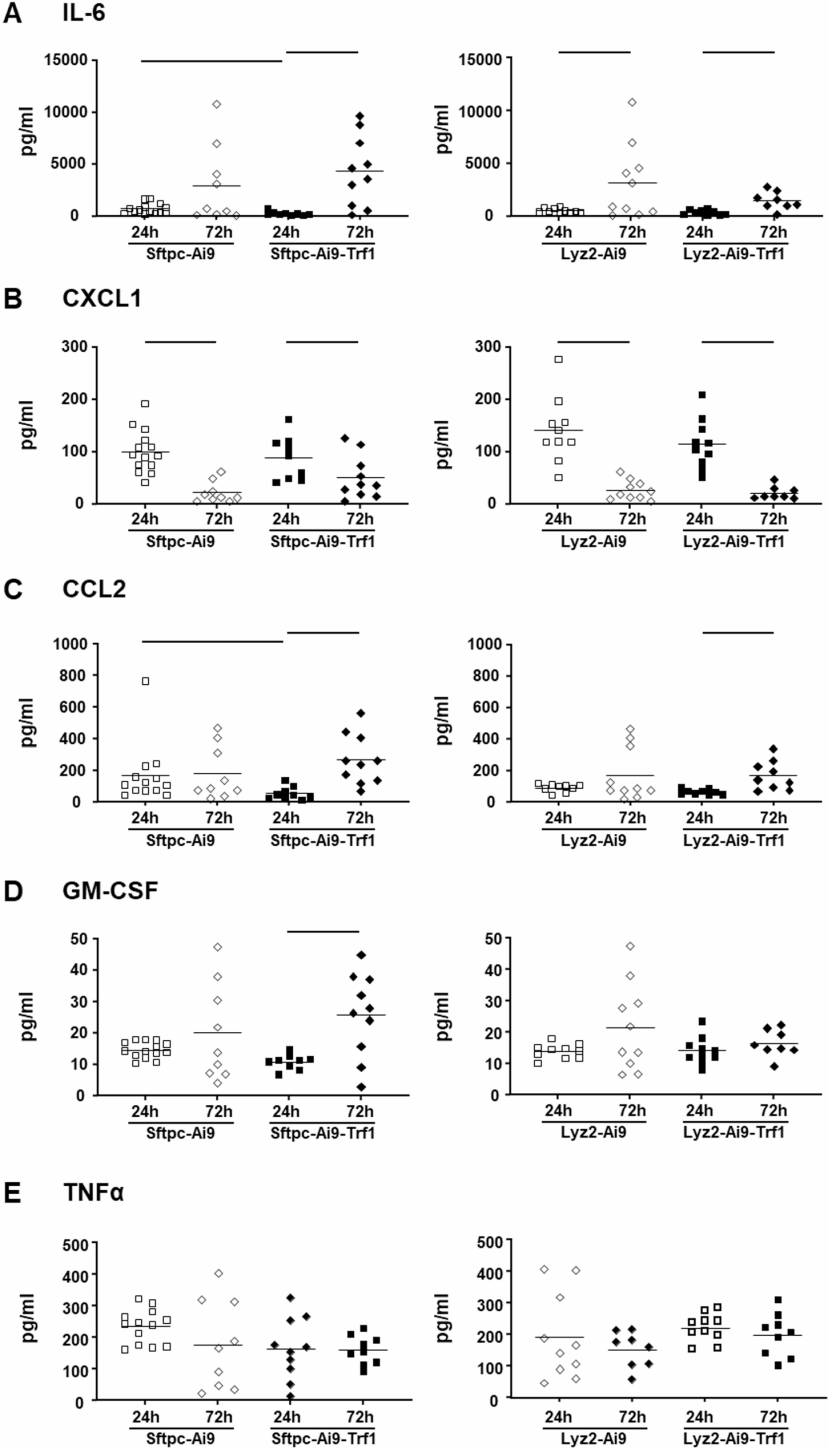
Cytokine expression in bronchoalveolar lavage fluid. Cytokine levels for IL-6 (**A**), CXCL1 (**B**), CCL2 (**C**), GM-CSF (**D**) and TNFα (**E**) were measured in BALF from LPS-treated Sftpc-Cre and Lyz2-Cre mice using LEGENDplex™. Only LPS-treated samples were analyzed due to assay detection limits. Statistical analysis was performed for Sftpc-Cre mice or Lyz2-Cre mice by two-way ANOVA with Bonferroni’s post hoc test to compare exposure and strain effects. Lines indicate significant differences between experimental groups (p< 0.05); *n* = 11-14 mice per LPS groups (each data point represents one animal)

The inflammatory response in the lung was quantitatively determined by measuring BALF protein, as an indicator of pulmonary edema, as well as BALF cytokine levels and cytometry. Induction of ALI by LPS exposures led to alveolar edema and pulmonary inflammation with elevated BALF protein and cells (Fig. 3), and cytokine levels (Fig. 4) in all strains.

In the Sftpc-Cre mice, the total number of BALF cells, neutrophils and macrophages was significantly higher in mice with Trf1 deletion 72 h after LPS administration compared to those without deletion (Fig. 3). In contrast, the Lyz2-Ai9-Trf1 mice showed a significantly higher increase in total BALF cells 24 h after LPS exposure than the corresponding mice without deletion (Fig. 3B). However, this increase in total BALF cells disappeared 72 h after induction of ALI.

The levels of BALF cytokines IL-6, CCL2 and GM-CSF in Sftpc-Ai9-Trf1 mice were further elevated after 72 h compared to 24 h of LPS treatment, which was not the case in the Sftpc-Ai9 mice (Fig. 4). Further Th1 cytokines such as TNFα, Il1β, and IFNγ were not differently regulated with Trf1 deletion (supplemental figure S2) and there were no significant differences in cytokine levels between Lyz2-Ai9-Trf1 and Lyz2-Ai9 mice. Altogether, this indicates a more severe inflammation in the late exudative phase of ALI with Trf1 deletion only in AEII cells and highlights the central role of AEII cell senescence in this model.

As concentration of BALF cytokines in control animals is below detection limit of the assay, we further compared cytokine expression in the lung tissue of control animals to test for primary signs of inflamm-aging due to Trf1 deletion. However, no signs of inflamm-aging were detectable in the lung tissue of Sftpc-Ai9-Trf1 or Lyz2-Ai9-Trf1 control mice. The results are presented in supplemental figure S3.

### Senescence response with Trf1 deletion in AEII cells and macrophages in ALI

To address the impact of Trf1 deletion on cellular senescence and cell cycle arrest in ALI, we analyzed the expression of established senescence markers, as well as the DDR and SASP, in whole lung tissue and specific cell population using scRNAseq in AEII cells, AMs and monocytes/interstitial macrophages (Mo/IMs). In addition, senescence-associated beta-galactosidase (SAβ-Gal) assay was performed on OCT embedded lung tissue section, however, the assay revealed no differences between experimental groups (methods and results are described in supplemental information and figure S4).

**Fig. 5:**
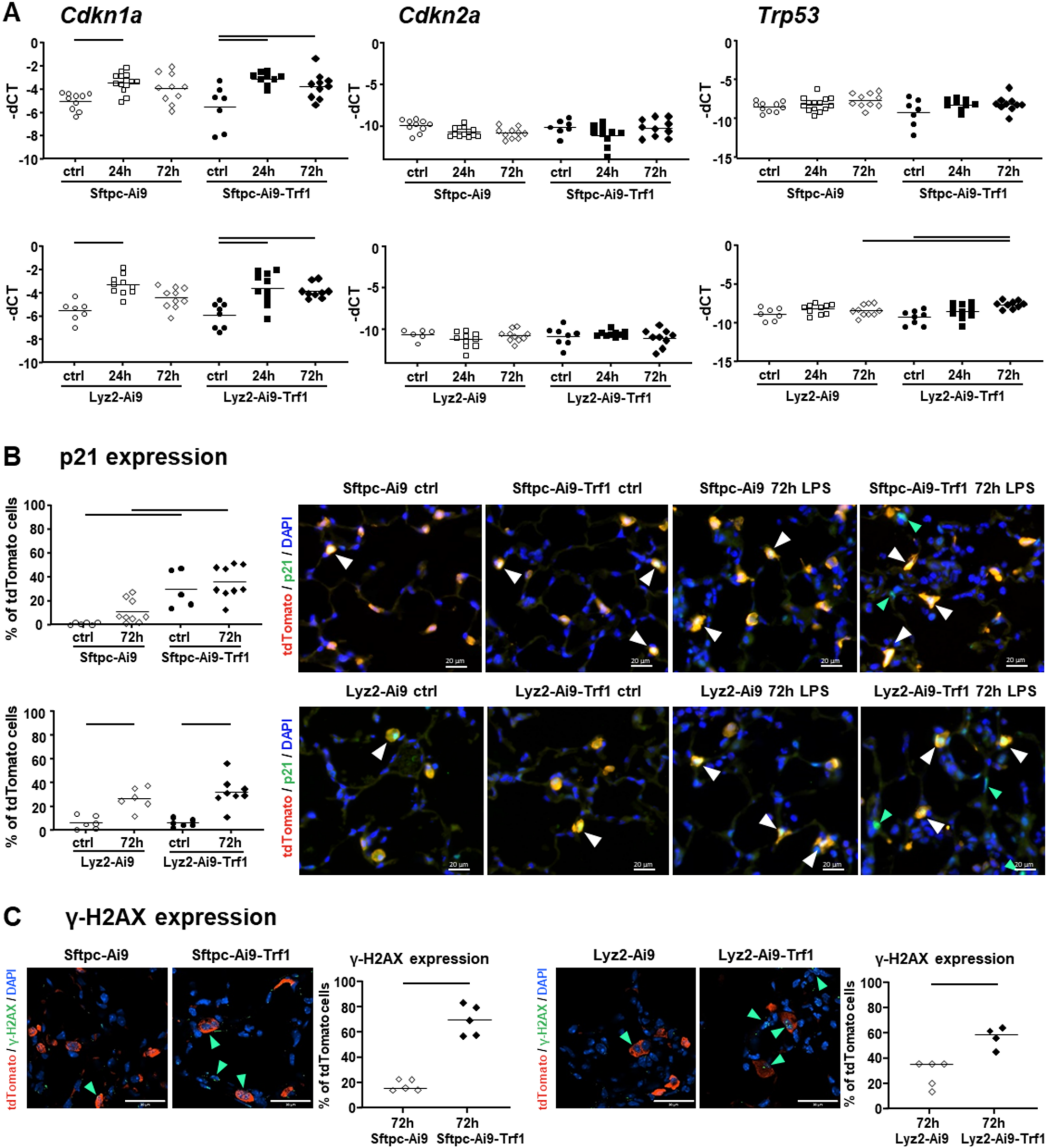
Gene expression analysis and immunofluorescence of senescence markers. Expression of senescence marker *Cdkn2a*, *Cdkn1a*, and *Trp53* were measured by real-time PCR in lung tissue (**A**). Expression of p21 in AEII cells or macrophages was additionally analyzed by IF in lung tissue sections (**B**). DAPI (blue) was used to detect nuclei and tdTomato (red) to detect AEII cells in Sftpc-Cre mice and macrophages in Lyz2-Cre mice. The percentage of p21 (green) positive AEII cells or macrophages (tdTomato positive cells) was counted and two-way ANOVA with Bonferroni’s post hoc test was applied to compare exposure and strain effects. Lines indicate significant differences between experimental groups (*p* < 0.05); *n* = 6–8 mice per saline groups and *n* = 6–9 mice per LPS groups. Green arrows indicate cells that were only p21 positive and white arrows p21 tdTomato double positive cells; scale bars = 20 μm. The expression of γ-H2AX (green) in tdTomato positive cells (red) was further analyzed by IF in Sftpc-Cre and Lyz2-Cre mice 72 h after LPS exposure (**C**). Statistical analysis was performed by Student’s t-test to compare strain effects; *n* = 4–5 per groups and scale bars = 30 μm. Green arrows indicate cells that were γ-H2AX positive in nuclei. Each data point represents one animal and lines indicate statistically significant differences (*p* < 0.05)

Gene expression analysis of senescence markers (*Cdkn1a*, *Cdkn2a* and *Trp53*) was performed in whole lung tissue (Fig. 5A). In general, ALI led to an increase in gene expression of *Cdkn1a* 24 h after LPS exposure but not of *Cdkn2a* or *Trp53.* At 72 h after induction of ALI, *Cdkn1a* remained upregulated in both, Sftpc-Cre and Lyz-Cre mice with Trf1 deletion but not in mice without Trf1 deletion. The expression of *Trp53* was also increased 72 h after LPS, but only in Lyz2-Ai9-Trf1 mice. These findings suggest that Trf1 deficiency in AEII cells or macrophages does not lead to a widespread activation of senescence markers in whole lung tissue. However, it is linked to a continuous *Cdkn1a* expression in late exudative phase of ALI, together with a selective upregulation of *Trp53* in Trf1-deleted macrophages.

Protein expression of p21 was furthermore specifically analyzed in AEII cells and macrophages of Sftpc-Cre and Lyz2-Cre mice, respectively, by IF 72 h after induction of ALI (Fig. 5B). In Sftpc-Cre mice with Trf1 deletion a significant increase in p21 positive AEII cells was detected compared to mice without Trf1 deletion, independent of ALI exposure. In Lyz2-Cre mice, however, p21 signaling was significantly elevated in macrophages after 72 h of LPS exposure, independent of Trf1 deletion. In contrast, IF expression of γ-H2AX was significantly elevated with Trf1 deletion in AEII cells and macrophages of Sftpc-Cre and Lyz2-Cre mice 72 h after LPS exposure (Fig. 5E). Additional analysis of strain effects in p53 and γ-H2AX expression in Sftpc-Cre and Lyz2-Cre control mice showed an increase in p53 expression (supplemental figure S4) in macrophages of Lyz2-Ai9-Trf1 mice (*p* = 0.057) and γ-H2AX expression was significantly elevated in AEII cells of Sftpc-Ai9-Trf1 (Fig. 1D). This indicates an increase in markers of senescence and DDR with Trf1 deletion in Sftpc-Cre control mice and in both, Sftpc-Cre as well as Lyz2-Cre mice, with LPS treatment.

**Fig. 6:**
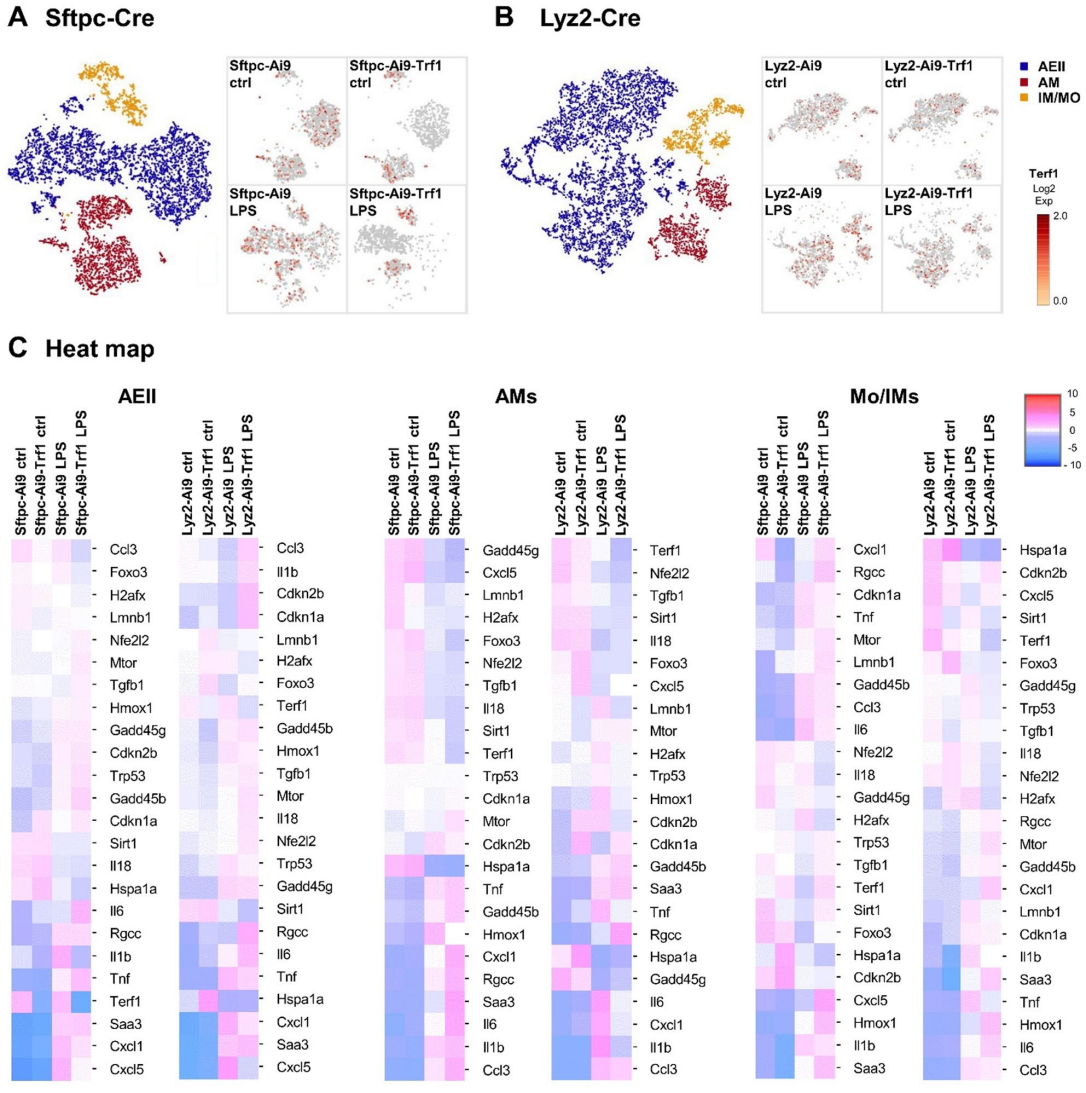
ScRNAseq analysis in AEII cells, AMs and Mo/IMs. Lung cell isolates from young Sftpc-Cre and Lyz2-Cre mice treated with either LPS (24 h exposure) or saline (sal) were uniquely labelled using oligo-barcodes. Pooled samples were clustered for clear classification and reclustered to focus on alveolar epithelial (AEII) cells, alveolar macrophages (AMs) and monocytes/interstitial macrophages (Mo/IMs). The t-SNE plots illustrate these specific cel types for the Sftpc-Cre (**A**) and Lyz2-Cre (**B**) mice. *Terf1* (Trf1) expression is also visualized in these populations. Furthermore heat-maps with gene expression profiles associated with markers for DDR and inflamm-aging were generated for AEII cells, AMs and Mo/IMs in Sftpc-Cre and Lyz2-Cre cell isolates (**C**)

In order to further assess cell type specific signaling of senescence, DDR and SASP in the Sftpc-Cre and Lyz2-Cre strains with and without Trf1 deletion, transcriptomics profiles of AEII cells, AMs and Mo/IMs were further compared using scRNAseq after 24 h of LPS or saline treatment (Fig. 6). Profiles of the analyzed cell populations and corresponding Trf1 expression are shown for experimental groups of Sftpc-Cre/Sftpc-Cre-Trf1 (Fig. 6A) and Lyz2-Cre/Lyz-Cre-Trf1 mice (Fig. 6B). In line with the observations from *Trf1* expression analysis and tdTomato IF, scRNAseq confirmed a AEII cell specific Trf1 deletion in Sftpc-Ai9-Trf1 mice (Fig. 6A and C). In Lyz2-Cre mice, *Trf1* expression was still present in AMs and Mo/IMs of Lyz2-Ai9-Trf1 mice (Fig. 6B and C), although to a lesser extent than in Lyz2-Ai9 mice. Even in AEII cells, a slight decline in *Trf1* expression was observed. This is also in line with the observation of a decreased Lyz2 promoter specificity shown in Fig. 1B.

Furthermore, heat maps (Fig. 6C) were used to illustrate the different gene expression profiles related to DDR, SASP and inflammation in the different cell populations of all experimental groups and its expression analysis, including statistical significance, is shown in the supplemental table S1-S6. In general, LPS treatment induced a significant upregulation of *Cxcl1*, *Cxcl5*, *Tnf*, *Saa3* (serum amyloid A 3) and *Rgcc* (regulator of cell cycle) in AEII cells of all strains. Upregulation of inflammatory markers and DDR was also observed in AMs after LPS exposure with increased expression of *Il1b*, *Ccl3*, *Saa3*, and *Rgcc* and a decrease in *Il18.* In Mo/IMs, *Saa3* and *Hmox1* (heme oxygenase 1) were elevated with LPS treatment in all strains.

In Sftpc-Cre mice, *Cdkn1a* was significantly more induced in AEII cells of controls with Trf1 deletion. Furthermore, a significant decline in *Trf1* was present in Sftpc-Ai9-Trf1 mice with and without LPS treatment, as discussed before. However, in AMs and Mo/IMs no significant differences in genes related to SASP/DDR were observed between mice with and without Trf1 deletion, independent of treatment groups. This indicates that Trf1 deletion in AEII cells led to a specific cell cycle inhibition, however, this did not promote inflammation or SASP in the early phase of ALI.

ScRNAseq of Lyz2-Cre mice also revealed signs of DDR and senescence in mice with Trf1 deletion, however, mostly only with LPS exposure. AEII cells from control mice showed an increase in *Rgcc* and *Hspa1a* (heat shock protein 1 A) in Lyz2-Ai9-Trf1 mice compared to Lyz2-Ai9, and a mild but significant increase in *Cdkn1a*, *Cdkn2b* and *Rgcc*. Furthermore, a decline in *Cxcl5* was detected with LPS treatment. In the AMs of the control animals with Trf1 deletion, a significant elevation in *Cdkn1a* was observed. An additional increase in *Rgcc* and lower *Il1b* expression was measured with LPS treatment compared to LPS treated animals without Trf1 deletion. No strain-related differences were detected in the Mo/IMs. Nevertheless, the data indicates that Trf1 deletion also promotes DDR signaling, in both AEII cells and AMs of the Lyz2-Cre mice, even though no overall increase in inflammation was observed.

### Impact of Trf1 deletion in AEII cells versus global aging on development of ALI

**Fig. 7:**
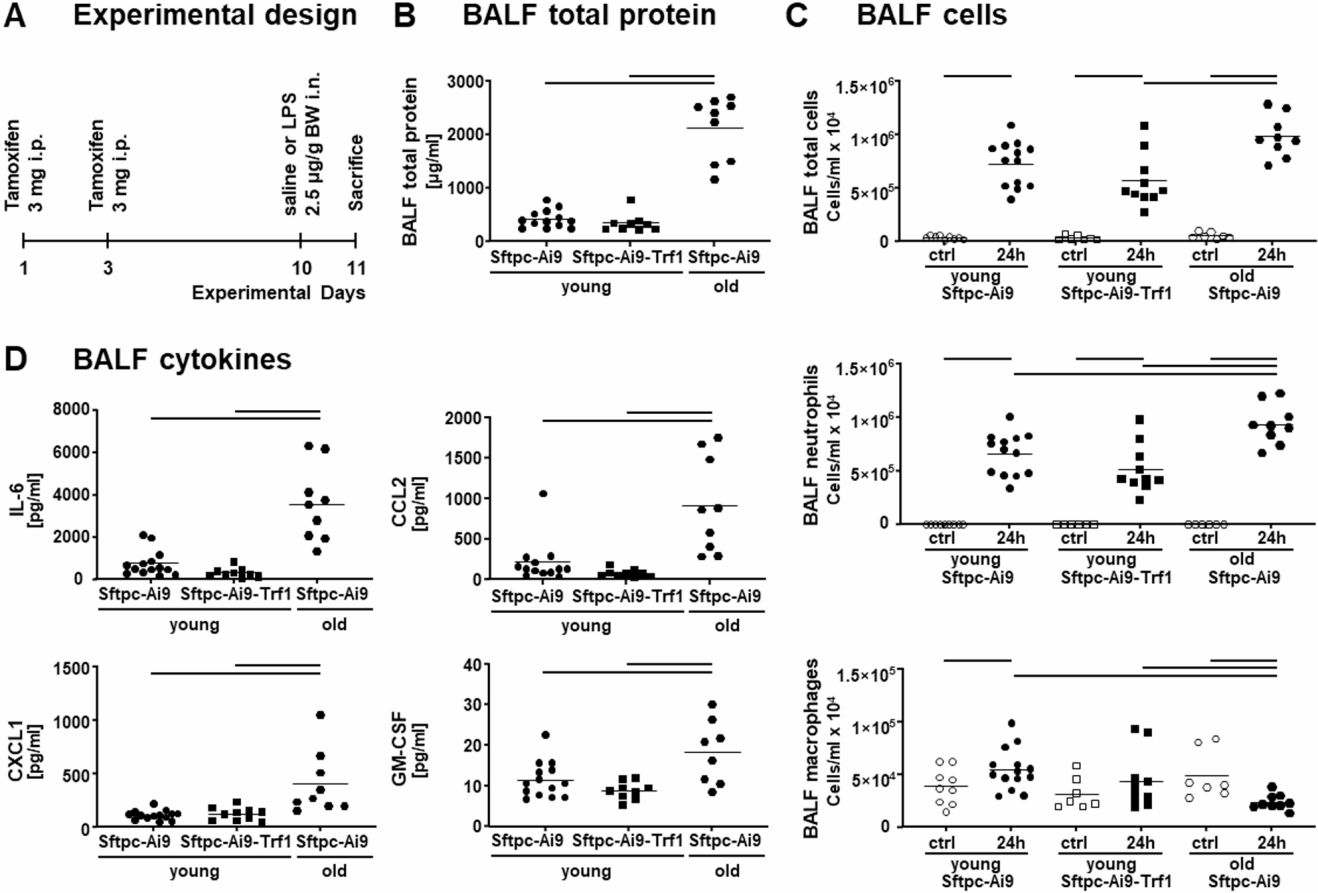
Comparison of AEII cell senescence and global aging. As shown in the experimental design (**A**), only the time point of 24 h LPS exposure was analyzed in old Sftpc-Ai9 mice and compared with young Sftpc-Ai9 and Sftpc-Ai9-Trf1 mice. The inflammatory response in the different experimental groups, was assessed by BALF protein (**B**), BALF cytometry (**C**), and BALF cytokines (**D**). Statistical analysis of BALF proteins and cytokines was performed by one-way ANOVA followed by Bonferroni’s post hoc test. For the other parameters, a two-way ANOVA followed by Bonferroni’s post hoc test was used. Lines indicate significant differences between experimental groups (*p* < 0.05); *n* = 8–10 mice per saline groups and *n* = 10–14 mice per LPS groups (each data point represents one animal)

To compare the enhanced inflammatory signaling caused by AEII cell senescence in the early exudative phase of ALI with the response in old mice, we also induced ALI in old (18 months) Sftpc-Ai9 mice. The experimental setup was the same as before and is depicted in Fig. 7A. Only the exposure time point of 24 h was analyzed, because previous studies showed severe age-related changes in ALI already after 24 h LPS [8, 19]. Comparing the results with the response in young (3 months) Sftpc-Ai9 and Sftpc-Ai9-Trf1 mice, ALI manifestation was much more severe in old mice with significantly increased BALF protein levels (Fig. 7B) and BALF cells (Fig. 7C). This provides evidence that age leads to a more pronounced recruitment of inflammatory cells to the lung during injury and, in line with this, also higher levels of BALF pro-inflammatory cytokines (IL-6, CXCL1, CCL2, GM-CSF; Fig. 7D). Hence, aging amplifies the inflammatory response in ALI and promotes injury progression, while Trf1-induced AEII cell senescence only affected inflammatory signaling in the late exudative phase of ALI and could not replicate the effects of global aging in ALI.

## Discussion

Cellular senescence has been proposed as a potential contributor to the pathogenesis of ALI in the elderly. However, specific populations of senescent cells and their potential role in the exacerbation of ALI have not yet been identified. In the current study, we therefore tested the hypothesis that telomere damage-mediated cellular senescence in different cell types, namely AEII cells and macrophages, contributes to the acute inflammatory response in ALI. Our findings suggest that experimentally induced senescence by Trf1 deletion in macrophages has no significant effect on the development of ALI, but senescence in AEII cells aggravates LPS-induced ALI in mice.

### The role of AEII cell senescence in ALI

In previous studies, we have shown that ALI in old mice causes senescence in AEII cells [7] and that AEII cell senescence impairs their proliferation in regeneration after injury [23]. This study advances our understanding of the role of AEII cell senescence in ALI by demonstrating that Trf1 deletion not only impairs regenerative proliferation, but also significantly contributes to inflammation and exacerbation in the late acute phase of ALI. This was manifested by an increase in tissue resistance (G) and elastance (H) as well as more inflammatory cells in the BALF of Sftpc-Ai9-Trf1 compared to Sftpc-Ai9 mice 72 h after LPS exposure. A significant elevation of BALF cytokines, namely IL-6, CCL2 and GM-CSF, from 24 h to 72 h after LPS treatment was furthermore found in Sftpc-Ai9-Trf1 mice, but not in the mice without Trf1 deletion. An increase in these parameters in ALI has been associated with a greater severity and the formation of septal edema [8, 31], confirming that Trf1 deletion-mediated senescence in AEII cells does contribute to the severity of ALI.

Considering that AEII cells are the progenitor cells of AEI cells and compensate to some extent their loss in the blood-air barrier in response to injury [22, 28, 32], it is reasonable to assume that AEII cell senescence could affect barrier integrity and support the development of edema. However, in previous studies with similar LPS models of ALI, AEII cell proliferation and AEI cell differentiation occurred only at later stages of regeneration (> 4 days post injury) [23, 33]. Therefore, it seems unlikely that limited AEII cell proliferation was responsible for the increased severity of ALI. Furthermore, no increase in apoptosis in AEII cells has been observed in the context of Trf1 deletion or advanced age in this LPS model of ALI [7, 23]. Consequently, it is more likely that alternative mechanisms such as SASP, inflammatory mediators, or DDR in senescent AEII cells contributed to the severity of ALI.

ScRNAseq data revealed that beside a strong decline in *Trf1* expression in AEII cells of Sftpc-Ai9-Trf1 mice, *Cdkn1a* expression was elevated. This finding was also confirmed by IF, showing an elevated expression of p21 in AEII cells of Sftpc-Ai9-Trf1 mice. In addition, an increase in γ-H2AX expression with Trf1 deletion was measured in AEII cells of Sftpc-Cre mice. However, no signs of increased inflammation or SASP were detectable in any of the cell types solely by Trf1 deletion. Previous proteome analysis of whole lung tissue of control mice with and without Trf1 deletion did also not reveal any type of SASP or inflamm-aging in Sftpc-Ai9-Trf1 mice [23]. Others have shown that long-term deletion of *Trf1* or *Trf2* in AEII cells over 3 months or longer is associated with the development of fibrosis and an increased susceptibility and mortality in injury models of bleomycin or influenza [26, 28, 30]. This is likely to be associated with the chronic effects of the Trf1/2 deletion with impaired proliferation and the enhanced severity of injury models compared to the LPS model. In our case, cell type-specific DDR was observed, as for example elevated γ-H2AX expression in AEII cells of Sftpc-Ai9-Trf1 mice with LPS exposure, but this was not associated with an elevation in SASP or inflamm-aging that could have potentially promoted enhanced severity in ALI. Consequently, any effects of AEII telomere damage-mediated cellular senescence may be relatively subtle in the short term and are likely to be triggered only by alterations in cellular signaling in response to injury. This hypothesis is supported by the observation that at the baseline and in the early stages of injury, no significant differences were evident between the mice with and without Trf1 deletion. Only at the subsequent exudative phase, differences in the severity of ALI became apparent in the Sftpc-Ai9-Trf1 mice. However, the underlying mechanisms remain to be resolved.

### Development of ALI in old mice

In contrast to Sftpc-Ai9-Trf1 mice showing increased signs of injury in the later phase of ALI only, old mice with regular Trf1 expression demonstrated increased lung injury/ inflammation in the early phase of ALI. This was evidenced by increased lung infiltration of immune cells and higher levels of pro-inflammatory cytokines compared to young mice with or without Trf1 deletion [7, 34, 35]. In addition, old mice showed signs of inflamm-aging prior to LPS exposure [23], such as increased alveolar macrophage activity, elevated basal cytokine levels and increased neutrophil recruitment, even in the absence of acute injury [6, 23, 36]. These might support the susceptibility of ALI development as suggested as well for elderly patients with ARDS [37, 38]. Furthermore, age-related changes in mitochondrial function and metabolic regulation of pulmonary immune cells have been demonstrated to impair inflammatory signaling after lung injury [36]. The control Sftpc-mice with Trf1 deletion, however, did not show elevated levels of pro-inflammatory cytokines (supplemental figure S3) or neutrophil numbers (Fig. 3) in their lung tissue, hence inflamm-aging was not detectable in young Sftpc-Ai9-Trf1 mice prior to LPS exposure. Notably though, in AEII cells of old control mice no signs of cellular senescence or DDR were present [7]. Taken together with the results of the previous studies [23], AEII cell senescence, as induced by Trf1 deletion, contributes to inflammation and severity of ALI but is not the primary cause of the pathogenesis and high mortality as observed in old age. Other cellular or systemic factors of aging such as inflamm-aging or a synergistic combination of various factors, are suggested to play a crucial role in the development of the disease in the elderly [6, 23, 36]. Nevertheless, our data shows that an altered cellular signaling of senescent AEII cells supports the inflammatory response in LPS-driven ALI and possibly its exacerbation.

### The role of macrophages with telomere damage-associated senescence in ALI

Given the central role of lung macrophages in the development of ALI [39–41], this study further focused on the effect of Trf1 deletion in macrophages from Lyz2-Cre mice. However, the deletion of *Trf1* in these mice did not significantly alter the severity or inflammatory response in LPS-induced ALI, nor did it induce significant inflammatory signaling in Lyz2-Ai9-Trf1 control mice (see supplemental figure S3).

The quantitative evaluation of Lyz2 promoter specificity has shown to have approximately 70% specificity for lung macrophages. Furthermore, off-target effects were observed in AEII cells which also express *Lyz2*. This has been confirmed as well with scRNAseq, showing a relatively high expression of *Lyz2* in AEII cells [29]. In contrast, the Sftpc promoter-related activation of the Cre-recombinase was very specific for AEII cells, as also shown by scRNAseq data. Consequently, it should be considered that limited efficiency of the Lyz2 promoter and expression in AEII cells may influence the interpretation of inflammatory responses. It remains elusive if a model with increased promoter specificity and/or prolonged tamoxifen exposure would have caused an exacerbation in ALI, similarly as in the Sftpc-Ai9-Trf1 mice.

Despite this limitation, examination of senescence markers in whole lung tissue and individual cell populations by scRNAseq showed signs of cell cycle arrest and DDR in Lyz2-Ai9-Trf1 mice with LPS exposure. As in Sftpc-Cre mice, Trf1 deletion resulted in a consistent increase in *Cdkn1a* expression in whole lung tissue 72 h after induction of ALI, which was not the case in animals without Trf1 deletion. Similarly, an increase of *Trp53* expression was detected in the lung tissue of 72 h LPS Lyz2-Ai9-Trf1 mice. In line with this, Lyz2-Ai9-Trf1 also showed a higher expression of histone γ-H2AX in macrophages of Lyz2-Ai9-Trf1 mice compared to mice without Trf1 deletion. The upregulation of these parameters is closely related to DDR mediated cell cycle arrest, and hence cellular senescence [42, 43]. This suggests an increase in DDR in ALI of the Lyz2-Cre mice with Trf1 deletion. In depth analysis with scRNAseq furthermore confirmed DDR response in AMs and AEII cells of the Lyz2-Ai9-Trf1 control mice and LPS-treated mice, i.e. in particular an increase in *Rgcc* and *Cdkn1a*. RGCC has been described as a transcriptional target of TRP53 and hence regulation of cell cycle arrest [44]. Despite this detection of senescence markers in AMs and AEII cells, no aggravation in inflammation and ALI was observed in the Lyz2-Cre mice with Trf1 deletion. This is in contrast to previous reports of AMs in old mice that are more responsive to inflammatory stimuli, such as LPS or bacterial infection, than those of young mice [19, 36, 45]. The increased activation of macrophages observed with age may therefore be due to an altered microenvironment in older individuals, i.e. inflamm-aging, rather than telomere/DDR-related cell senescence. However, an increase in SASP or inflamm-aging was not present in mice with Trf1 deletion. Thus, other than initially hypothesized, Trf1 deletion in Lyz2-Cre mice did not induce robust signs of senescence in macrophages and does not seem to be a substantial contributor to inflamm-aging and ALI in the described model.

## Conclusions

The data of our study show that senescence and DDR in AEII cells aggravates the development of ALI. However, DDR and cellular senescence in lung cells are only partly mimicking the phenotype of aging and telomere damage-induced senescence did not reflect the full spectrum of aging and enhanced severity in old mice. This suggests that the general aging process, possibly also driven by inflamm-aging, has a more profound impact on the pathogenesis of the disease. Therefore, interventions targeting cellular senescence should not only focus on specific cell types but also consider the broader aspects of aging. Future research should further elucidate the contributions of the mechanisms of aging to gain a holistic understanding and develop effective therapeutic interventions for ALI in the elderly.

## Supplementary Information

Supplementary Material 1.

Supplementary Material 2.

Supplementary Material 3.

## Data Availability

ScRNAseq data will be made publicly available via GEO upon acceptance of the manuscript. Any other raw data that is not included in the paper or supplemental information will be made available on request.

## Abbreviations

AEI cells: Alveolar Epithelial Type I Cells
AEII cells: Alveolar Epithelial Type II Cells
ALI: Acute Lung Injury
AM: Alveolar Macrophages
ARDS: Acute Respiratory Distress Syndrome
BALF: Bronchoalveolar Lavage Fluid
CCL2: CC-chemokin-ligand 2
Cdkn1a (p21): Cyclin Dependent Kinase Inhibitor 1 A
Cdkn2a (p16): Cyclin Dependent Kinase Inhibitor 2 A
Cst: Compliance
CXCL1: C-X-C motif chemokine ligand 1
DDR: DNA Damage Response
G: Tissue Resistance
GM-CSF: Granulocyte-Macrophage Colony-Stimulating Factor
H: Elastance
Hmox1: Heme Oxygenase 1
Hprt1: Hypoxanthin-Phosphoribosyl-Transferase 1
Hspa1a: Heat Shock Protein 1 A
IF: Immunofluorescence
IL: Interleukin
LPS: Lipopolysaccharide
Lyz2: Lysozyme 2
Lyz2-Ai9: B6.Cg-Lyz2^tm1(cre/ERT2)Grtn^ Gt(ROSA)26Sor^tm9(CAG−tdTomato)Hze^
Lyz2-Ai9-Trf1: B6.Cg-Lyz2^tm1(cre/ERT2)Grtn^ Gt(ROSA)26Sor^tm9(CAG−tdTomato)Hze^ Terf1^tm2.1Tdl^
Mo/Ims: Monocytes/Interstitial Macrophages
PFA: Paraformaldehyde
Rgcc: Regulator of Cell Cycle
Saa3: Serum Amyloid A 3
SASP: Senescence Associated Secretory Phenotype
scRNAseq: Single-cell RNA sequencing
Sftpc: Surfactant Protein C
Sftpc-Ai9: B6.Cg-Sftpc^tm1(cre/ERT2)Blh^ Gt(ROSA)26Sor^tm9(CAG−tdTomato)Hze^
Sftpc-Ai9-Trf1: B6.Cg-Sftpc^tm1(cre/ERT2)Blh^ Gt(ROSA)26Sor^tm9(CAG−tdTomato)Hze^ Terf1^tm2.1Tdl^
Tnf: Tumor Necrosis Factor Alpha
Trf1: Telomeric Repeat-Binding Factor 1
Trp53 (p53): Tumor Protein 53
γ-H2AX: Gamma H2A Histone Family Member X
