# Supplementary material for: Telomere damage-mediated senescence in alveolar epithelial type II cells but not in macrophages aggravates inflammation in acute lung injury
Source: Respir Res. 2026 Mar 14;27:161. doi: 10.1186/s12931-026-03627-0 (PMC13063740; doi:10.1186/s12931-026-03627-0)
Supplement: Supplementary file 1 — Supplementary Material 1. [file 12931_2026_3627_MOESM1_ESM.pdf]

Supplemental Information

Telomere damage-mediated senescence in alveolar epithelial type II cells but not in macrophages aggravates inflammation in acute lung injury

Christina B. Hildebrand, Seyma Öztürk, Jia Li Ye, Dirk Wedekind, Ulrich A. Maus, Christian Bär, Christian Mühlfeld, Christina Brandenberger

Breeding Strategy of animals

The induction of telomere damage in AEII cells and macrophages was achieved by generating corresponding transgenic mouse lines. Trf1 deletion was achieved by expressing Cre recombinase under the Sftpc promoter in AEII cells and under the Lyz2 promoter in macrophages. While the Lyz2 promoter is primarily expressed by macrophages, it is also present in other myeloid cells, including monocytes and neutrophils. To visualize Cre recombinase activity, a mouse strain was bred that exhibits Cre-inducible expression of tdTomato, a red fluorescent protein (Ai9). The experiments were conducted using four different mouse strains, which were mated according to the schemes shown in Supplemental Figure S1 and as previously described (1): Sftpc-Ai9 (B6.Cg-Sftpctm1(cre/ERT2)Blh Gt(ROSA)26Sortm9(CAG-tdTomato)Hze), Sftpc-Ai9-Trf1 (B6.Cg-Sftpctm1(cre/ERT2)Blh Gt(ROSA)26Sortm9(CAG-tdTomato)Hze Terf1tm2.1Tdl), Lyz2-Ai9 (B6.Cg-Lyz2tm1(cre/ERT2)Grtn Gt(ROSA)26Sortm9(CAG-tdTomato)Hze) and Lyz2-Ai9-Trf1 (B6.Cg-Lyz2tm1(cre/ERT2)Grtn Gt(ROSA)26Sortm9(CAG-tdTomato)Hze Terf1tm2.1Tdl) mice.

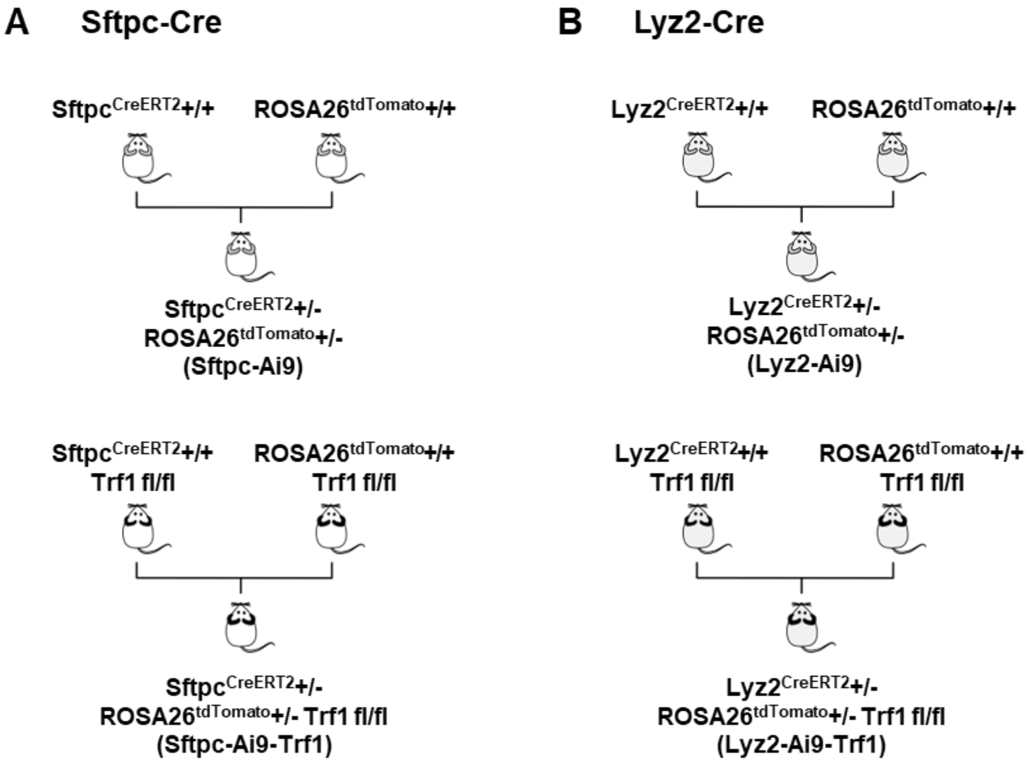

Supplemental Figure S1. Transgenic mouse model. Transgenic mouse lines were generated as shown in schemes (A) for Sftpc-Cre and (B) Lyz2-Cre mice, respectively, with and without Trf1 deletion.

Th1 inflammatory cytokines in BALF

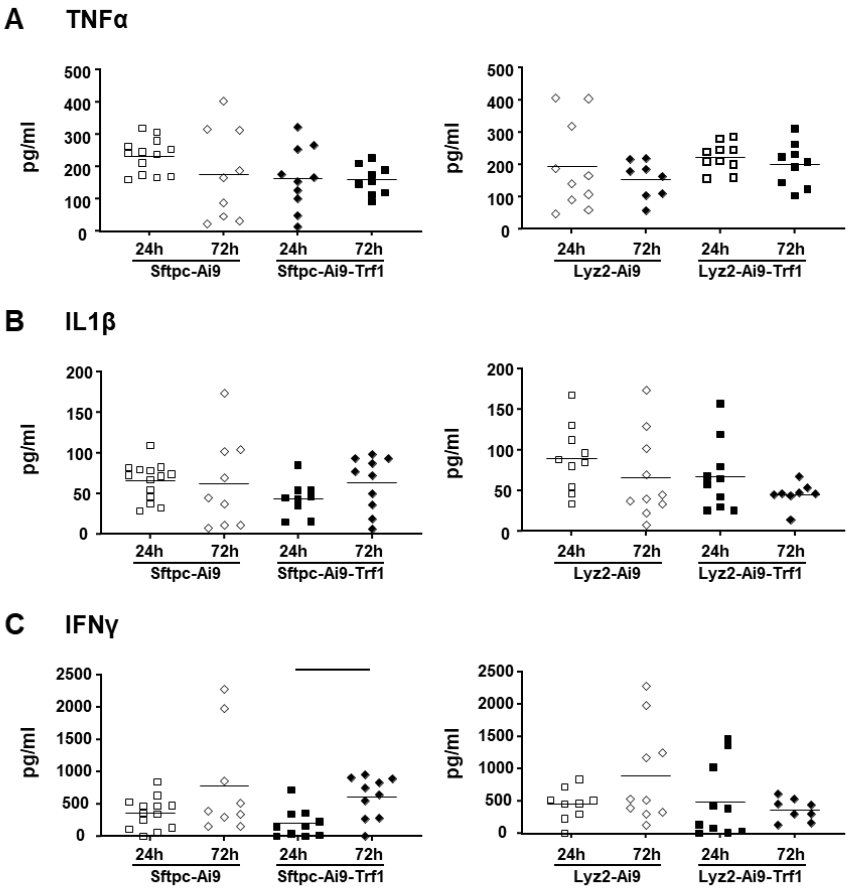

Supplemental Figure S2. Additional Th1 inflammation markers in bronchoalveolar lavage fluid. Cytokine levels for TNFα (A), IL1β (B), and IFNγ (E) were measured in BALF from LPS-treated Sftpc-Cre and Lyz2-Cre mice. No significant differences were observed between mice with and without Trf1 deletion. Statistical analysis was performed for Sftpc-Cre mice or Lyz2-Cre mice by two-way ANOVA with Bonferroni's post hoc test to compare exposure and strain effects. Lines indicate significant differences between experimental groups ($p < 0.05$); n = 11-14 mice per LPS groups (each data point represents one animal).

Cytokine expression in lung tissue

Cytokine concentrations were determined in the lung tissue of control Sftpc-Cre and Lyz2-Cre mice, as described previously (2). In brief, the snap frozen middle lung lobe was immersed in protease inhibitor (Complete Tablets Mini, Roche) containing RIPA buffer

(Thermo Scientific) and homogenized using a Tissue Lyser (Qiagen). Cell debris was removed by centrifugation at 21000 g for 15 min at 4°C. Supernatant was used for cytokine quantification using the 13-plex cytokine bead array (LEGENDplex™, BioLegend) according to the manufacturer`s instructions. Cytokine concentrations were further normalized to total protein content, determined with the BCA Protein Assay Kit (Thermo Scientific).

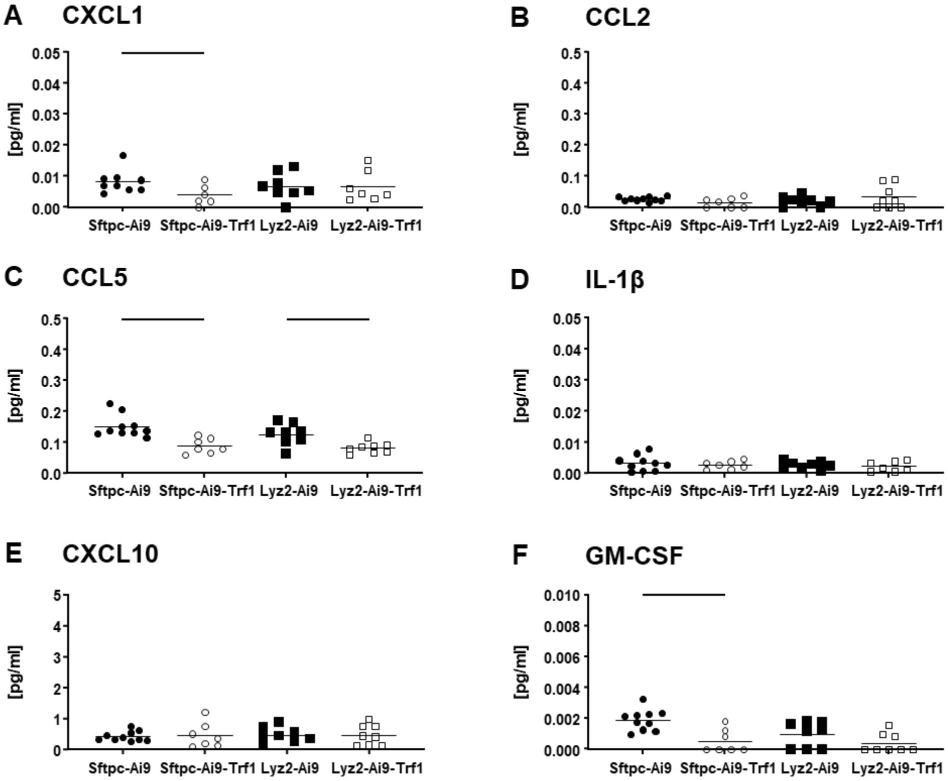

Supplemental Figure S3. Cytokine expression in lung tissue were measured for CXCL-1 (A), CCL2 (B), CCL5 (C), IL-1β (D), CXCL10 (E) and GM-CSF (F). A decline in CCL5 expression was observed in both mouse lines with Trf1 deletion, as well as in CXCL1 and GM-CSF in Sftpc-Ai9-Trf1 mice. Inflammatory cytokines such as IL-6 or TNFα are not displayed due to assay detection limits. Statistical analysis was performed by one-way ANOVA with Bonferroni’s post hoc test to compare strain effects in Sftpc-Cre and Lyz2-Cre mice. Lines indicate significant differences between experimental groups ($p < 0.05$); n = 8-10 mice per group.

Senescence-associated β-Galactosidase activity staining protocol and results

Senescence-associated β-Galactosidase (SAβ-Gal) staining was performed based on manufacture's instruction (#9860S, Cell Signaling Technology) and the staining protocol from Idelfonso-García et al. (3) with some modifications. In brief, OCT embedded lung tissue was sectioned into 5 μm thick slices. Sections were incubated for 48 hours at 37 °C incubator with the staining solution. At the end of incubation, slides were washed three times with PBS and mounted in Mowiol. The slides were scanned with AxioScan.Z1 at 20x magnification (Zeiss). Approximately 30 images per lung tissue section were sub-sampled with systematic uniform random sampling (SURS) at 20x magnification using the Visiopharm software (Visiopharm, Hørsholm, Denmark) to secure unbiased image sampling. Afterwards, image analysis was performed based on Krzystyniak et al. Fiji-based macro extension and the blue stained area was detected and normalized to total area of section (4). In addition, tdTomato positive cells were analysed for co-expression of SAβ-Gal in control mice. Therefore, the images were acquired with a fluorescence microscope (BZ-X800, Keyence) at a 20x magnification, combining bright-field and fluorescence imaging for the analysis. Cells were counted manually with a minimum of 200 tdTomato or SAβ-Gal positive cells in Sftpc-Cre and Lyz2-Cre mice with a 3x digital zoom. Results show percentage of SAβ-Gal-stained tissue sections, as well as the percentage of tdTomato positive cells with a positive SAβ-Gal signal. However, no differences in SAβ-Gal staining were observed between strains with and without Trf1 deletion. Therefore, it should be noted that SAβ-Gal activity is not an exclusive marker of senescence in vivo (5), and may be elevated in macrophages independently of the senescent phenotype (6). The high levels of SAβ-Gal activity exhibited in macrophages as observed in our study as well, may be rather due to the large number and size of their lysosomes (7). Furthermore, previous data from a comparable SPC-Cre Trf1(fl/fl) model suggest that a robust SAβ-Gal signal in AEII cells only becomes evident following prolonged Trf1 deletion (8 months), while shorter deletion periods showed DNA damage without a robust SAβ-Gal signal (8), which is in line with our findings.

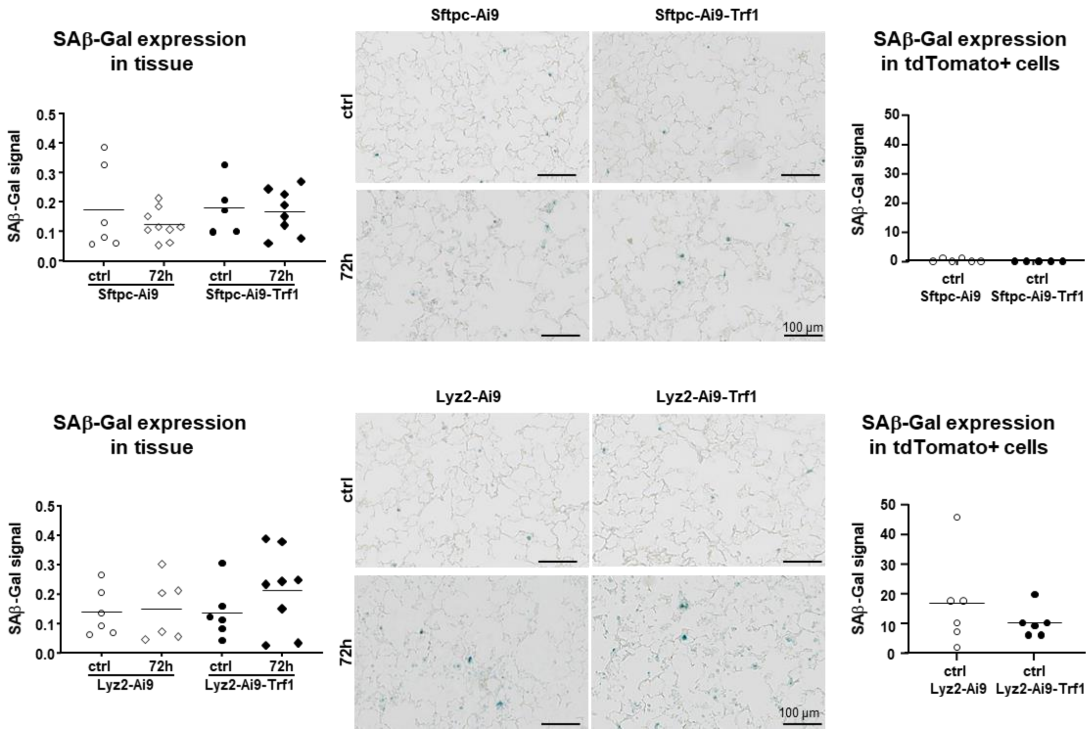

Supplemental Figure S4. SAβ-Gal staining in total lung tissue and tdTomato positive cells. The percentage of the blue SAβ-Gal positive staining signal was assessed in the lung tissue of Sftpc-Cre and Lyz2-Cre mice under control conditions and 72 h after LPS exposure. Additionally, the percentage of SAβ-Gal and tdTomato double positive cells was assessed in control mice. Statistical analysis was performed using two-way or one-way ANOVA followed by Bonferroni's post hoc test, assessing both LPS treatment effects and strain effects. However, no significant differences in SAβ-Gal expression were observed between strains or with LPS treatment. Representative images illustrate very similar SAβ-Gal signal (blue) across the different experimental groups.

P53 staining protocol and results

Analogous to the p21 staining protocol described in methods section, lung tissues from saline treated Sftpc-Cre and Lyz2-Cre mice were stained with rabbit anti-p53 antibody (ab246550, Abcam) at a dilution of 1:500. Stained sections were scanned at a 20x magnification with an AxioScan.Z1 scanner (Zeiss). At least 200 tdTomato positive cells were counted and the percentage of p53 positive double positive cells was determined.

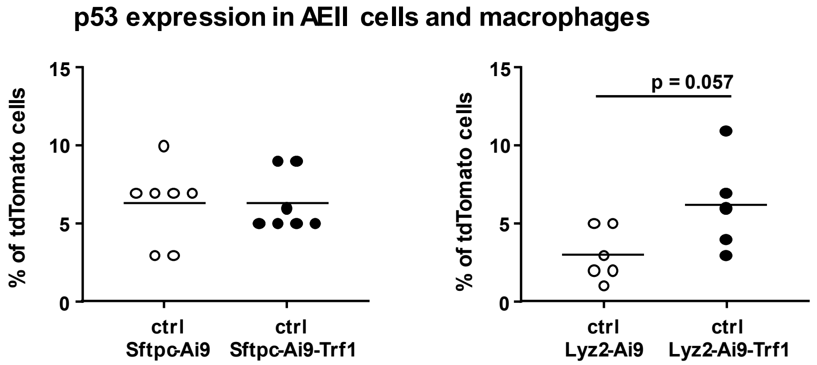

Supplemental Figure S5. Expression of p53 in control Sftpc-Cre and Lyz2-Cre mice was assessed with immunofluorescence. Percentage of p53 positive AEII cells or macrophages were counted and one-way ANOVA with Bonferroni's post hoc test was used to compare strain effects. No significant differences in p53 expression were observed between strains, but a tendency ($p = 0.057$) of an increase in Lyz2-Ai9-Trf1 mice. Each data point represents one animal, and lines indicate significant differences between experimental groups ($p < 0.05$); n = 6-8 mice per group.

References

1. Hirsch MS, Hildebrand CB, Geltinger F, Pich A, Mühlfeld C, Wedekind D, et al. Senescence in alveolar epithelial type II cells promotes acute lung injury and impairs regeneration. Am J Respir Cell Mol Biol. 2024;71:688–701. https://doi.org/10.1165/rcmb.2024-0054OC

2. Hildebrand CB, Lichatz R, Pich A, Mühlfeld C, Woltemate S, Vital M, Brandenberger C. Short-chain fatty acids improve inflamm-aging and acute lung injury in old mice. American Journal of Physiology-Lung Cellular and Molecular Physiology (2023) 324:L480–L492.

3. Idelfonso-García OG, Pacheco-Rivera R, Alarcón-Sánchez BR, Serrano-Luna J, Baltiérrez-Hoyos R, Vásquez-Garzón VR, Muriel P, Villa-Treviño S, Pérez-Carreón JI, Arellanes-Robledo J. Protocol to detect senescence-associated β-galactosidase and immunoperoxidase activity in fresh-frozen murine tissues. STAR Protoc. 2024 Jun 21;5(2):103009.

4. Krzystyniak, A.; Gluchowska, A.; Mosieniak, G.; Sikora, E. Fiji-Based Tool for Rapid and Unbiased Analysis of SA-β-Gal Activity in Cultured Cells. Biomolecules 2023, 13, 362.

5. González-Gualda E, Baker AG, Fruk L, Muñoz-Espín D. A guide to assessing cellular senescence in vitro and in vivo. FEBS J. 2021 Jan;288(1):56-80.

6. Deng X, Yin Z, Tai S, Wang Y, Fu L. Macrophage Senescence: Friend or Foe? Aging Dis. 2026 Feb 6

7. Ogrodnik M, Carlos Acosta J, Adams PD, d'Adda di Fagagna F, Baker DJ, Bishop CL, Chandra T, Collado M, Gil J, Gorgoulis V, Gruber F, Hara E, Jansen-Dürr P, Jurk D, Khosla S, Kirkland JL, Krizhanovsky V, Minamino T, Niedernhofer LJ, Passos JF, Ring NAR, Redl H, Robbins PD, Rodier F, Scharffetter-Kochanek K, Sedivy JM, Sikora E, Witwer K, von Zglinicki T, Yun MH, Grillari J, Demaria M. Guidelines for minimal information on cellular senescence experimentation in vivo. Cell. 2024 Aug 8;187(16):4150-4175

8. Naikawadi RP, Disayabutr S, Mallavia B, Donne ML, Green G, La JL, Rock JR, Looney MR, Wolters PJ. Telomere dysfunction in alveolar epithelial cells causes lung remodeling and fibrosis. JCI Insight. 2016 Sep 8;1(14):e86704
