# Supplementary material for: Telomere damage-mediated senescence in alveolar epithelial type II cells but not in macrophages aggravates inflammation in acute lung injury
Source: Respir Res. 2026 Mar 14;27:161. doi: 10.1186/s12931-026-03627-0 (PMC13063740; doi:10.1186/s12931-026-03627-0)
Supplement: Supplementary file 2 — Supplementary Material 2. [file 12931_2026_3627_MOESM2_ESM.docx]

Table S1: AEII cells – strain effects; significant differences with p < 0,1 are bold; significant upregulation is black and downregulation gray

| **Gene** | ctrl | | | LPS | | | ctrl | | | LPS | | |
| --- | --- | --- | --- | --- | --- | --- | --- | --- | --- | --- | --- | --- |
|  | Sftpc-Ai9 | Sftpc-Ai9-Trf1 | p-Value | Sftpc-Ai9 | Sftpc-Ai9-Trf1 | p-Value | Lyz2-Ai9 | Lyz2-Ai9-Trf1 | p-Value | Lyz2-Ai9 | Lyz2-Ai9-Trf1 | p-Value |
|  | Average | Average |  | Average | Average |  | Average | Average |  | Average | Average |  |
| *Ccl3* | 0,011 | 0,008 | 1 | 0,015 | 0,008 | 1 | 0,010 | 0,008 | 1 | 0,009 | 0,021 | 1 |
| *Cdkn1a* | 0,297 | 0,752 | **0,062** | 0,881 | 1,061 | 1 | 0,218 | 0,364 | 1 | 0,351 | 1,055 | **2,06×10^-06^** |
| *Cdkn2b* | 0,055 | 0,047 | 1 | 0,138 | 0,144 | 1 | 0,077 | 0,075 | 1 | 0,090 | 0,259 | **1,12×10^-04^** |
| *Cxcl1* | 0,011 | 0,021 | 1 | 2,295 | 1,701 | 1 | 0,019 | 0,050 | 1 | 3,778 | 2,147 | 0,196 |
| *Cxcl5* | 0,005 | 0,008 | 1 | 2,108 | 1,188 | 0,889 | 0,043 | 0,087 | 1 | 9,167 | 2,162 | **1,82×10^-11^** |
| *Foxo3* | 0,276 | 0,251 | 1 | 0,437 | 0,334 | 1 | 0,202 | 0,306 | 1 | 0,213 | 0,325 | 0,806 |
| *Gadd45b* | 0,132 | 0,184 | 1 | 0,505 | 0,639 | 1 | 0,254 | 0,157 | 1 | 0,490 | 0,526 | 1 |
| *Gadd45g* | 0,328 | 0,415 | 1 | 0,953 | 0,960 | 1 | 0,338 | 0,352 | 1 | 1,304 | 1,224 | 1 |
| *H2afx* | 0,062 | 0,059 | 1 | 0,087 | 0,078 | 1 | 0,054 | 0,065 | 1 | 0,090 | 0,066 | 1 |
| *Hmox1* | 0,294 | 0,327 | 1 | 0,651 | 0,677 | 1 | 0,264 | 0,212 | 1 | 0,577 | 0,516 | 1 |
| *Hspa1a* | 0,034 | 0,053 | 1 | 0,037 | 0,025 | 1 | 0,067 | 0,293 | **1,00×10^-08^** | 0,044 | 0,048 | 1 |
| *Il18* | 0,015 | 0,018 | 1 | 0,013 | 0,012 | 1 | 0,007 | 0,007 | 1 | 0,012 | 0,014 | 1 |
| *Il1b* | 0,014 | 0,005 | 1 | 0,062 | 0,013 | 1 | 0,012 | 0,011 | 1 | 0,011 | 0,022 | 1 |
| *Il6* | 0,001 | 0,003 | 1 | 0,007 | 0,018 | 1 | 8,33×10^-04^ | 0,005 | 1 | 0,016 | 0,027 | 1 |
| *Lmnb1* | 0,042 | 0,032 | 1 | 0,061 | 0,058 | 1 | 0,037 | 0,046 | 1 | 0,048 | 0,051 | 1 |
| *Mtor* | 0,076 | 0,074 | 1 | 0,133 | 0,155 | 1 | 0,080 | 0,075 | 1 | 0,131 | 0,168 | 1 |
| *Nfe2l2* | 0,848 | 0,951 | 1 | 1,445 | 1,666 | 1 | 0,925 | 1,049 | 1 | 1,589 | 1,965 | 1 |
| *Rgcc* | 1,285 | 1,502 | 1 | 8,484 | 8,363 | 1 | 0,652 | 1,797 | **1,10×10^-05^** | 2,393 | 9,198 | **1,91×10^-11^** |
| *Saa3* | 0,326 | 0,376 | 1 | 54,901 | 58,388 | 1 | 0,430 | 0,691 | 1 | 51,810 | 54,203 | 1 |
| *Sirt1* | 0,076 | 0,078 | 1 | 0,076 | 0,076 | 1 | 0,080 | 0,089 | 1 | 0,067 | 0,040 | 0,949 |
| *Terf1* | 0,063 | 0,000 | **4,93×10^-07^** | 0,077 | 0,000 | **1,29×10^-13^** | 0,052 | 0,037 | 1 | 0,082 | 0,046 | 0,593 |
| *Tgfb1* | 0,062 | 0,059 | 1 | 0,087 | 0,110 | 1 | 0,067 | 0,059 | 1 | 0,122 | 0,130 | 1 |
| *Tnf* | 0,001 | 0,000 | 1 | 0,027 | 0,042 | 1 | 0,002 | 0,002 | 1 | 0,038 | 0,028 | 1 |
| *Trp53* | 0,087 | 0,066 | 1 | 0,206 | 0,239 | 1 | 0,072 | 0,096 | 1 | 0,216 | 0,181 | 1 |

Table S2: AEII cells – LPS effects; significant differences with p < 0,1 are bold; significant upregulation is black and downregulation gray

| **Gene** | Sftpc-Ai9 | | | Sftpc-Ai9-Trf1 | | | Lyz2-Ai9 | | | Lyz2-Ai9-Trf1 | | |
| --- | --- | --- | --- | --- | --- | --- | --- | --- | --- | --- | --- | --- |
|  | ctrl | LPS | p-Value | ctrl | LPS | p-Value | ctrl | LPS | p-Value | ctrl | LPS | p-Value |
|  | Average | Average |  | Average | Average |  | Average | Average |  | Average | Average |  |
| *Ccl3* | 0,012 | 0,011 | 1 | 0,01 | 0,01 | 0,912 | 0,012 | 0,007 | 0,755 | 0,010 | 0,018 | 0,711 |
| *Cdkn1a* | 0,328 | 0,618 | **0,035** | 1,11 | 0,99 | 0,906 | 0,257 | 0,289 | 0,873 | 0,432 | 0,876 | **9,41×10^-04^** |
| *Cdkn2b* | 0,061 | 0,097 | 0,321 | 0,07 | 0,14 | 0,217 | 0,090 | 0,074 | 0,755 | 0,089 | 0,215 | **1,53×10^-04^** |
| *Cxcl1* | 0,012 | 1,610 | **1,55×10^-36^** | 0,03 | 1,59 | **2,41×10^-18^** | 0,023 | 3,114 | **1,79×10^-61^** | 0,059 | 1,784 | **1,24×10^-32^** |
| *Cxcl5* | 0,006 | 1,478 | **1,08×10^-31^** | 0,01 | 1,11 | **2,95×10^-13^** | 0,051 | 7,557 | **7,37×10^-70^** | 0,103 | 1,796 | **4,97×10^-25^** |
| *Foxo3* | 0,305 | 0,307 | 1 | 0,37 | 0,31 | 0,806 | 0,238 | 0,176 | 0,422 | 0,363 | 0,270 | 0,293 |
| *Gadd45b* | 0,146 | 0,354 | **0,001** | 0,27 | 0,60 | **0,033** | 0,299 | 0,404 | 0,399 | 0,187 | 0,437 | **9,53×10^-05^** |
| *Gadd45g* | 0,362 | 0,668 | **0,032** | 0,61 | 0,90 | 0,458 | 0,398 | 1,075 | **2,63×10^-06^** | 0,417 | 1,016 | **1,28×10^-05^** |
| *H2afx* | 0,069 | 0,061 | 0,900 | 0,09 | 0,07 | 0,884 | 0,064 | 0,075 | 0,837 | 0,077 | 0,055 | 0,451 |
| *Hmox1* | 0,324 | 0,457 | 0,368 | 0,48 | 0,63 | 0,665 | 0,311 | 0,476 | 0,126 | 0,252 | 0,429 | **0,025** |
| *Hspa1a* | 0,037 | 0,026 | 0,626 | 0,08 | 0,02 | **0,011** | 0,079 | 0,037 | **0,017** | 0,348 | 0,040 | **1,56×10^-22^** |
| *Il18* | 0,016 | 0,009 | 0,639 | 0,03 | 0,01 | 0,384 | 0,009 | 0,010 | 1 | 0,008 | 0,012 | 0,790 |
| *Il1b* | 0,015 | 0,044 | 0,551 | 0,01 | 0,01 | 0,844 | 0,014 | 0,009 | 0,785 | 0,013 | 0,018 | 0,706 |
| *Il6* | 0,001 | 0,005 | 0,571 | 0,00 | 0,02 | 0,455 | 0,001 | 0,013 | **0,012** | 0,006 | 0,023 | **0,011** |
| *Lmnb1* | 0,047 | 0,043 | 0,958 | 0,05 | 0,05 | 1 | 0,044 | 0,039 | 0,923 | 0,055 | 0,042 | 0,575 |
| *Mtor* | 0,084 | 0,093 | 0,949 | 0,11 | 0,15 | 0,730 | 0,094 | 0,108 | 0,840 | 0,089 | 0,139 | 0,152 |
| *Nfe2l2* | 0,936 | 1,013 | 0,955 | 1,40 | 1,56 | 0,972 | 1,089 | 1,310 | 0,661 | 1,245 | 1,632 | 0,323 |
| *Rgcc* | 1,419 | 5,950 | **3,85×10^-11^** | 2,22 | 7,84 | **9,02×10^-06^** | 0,767 | 1,973 | **4,65×10^-06^** | 2,133 | 7,639 | **1,09×10^-11^** |
| *Saa3* | 0,359 | 38,503 | **2,34×10^-48^** | 0,55 | 54,70 | **2,33×10^-31^** | 0,506 | 42,713 | **2,08×10^-66^** | 0,820 | 45,017 | **1,04×10^-57^** |
| *Sirt1* | 0,084 | 0,053 | 0,275 | 0,12 | 0,07 | 0,368 | 0,094 | 0,055 | 0,111 | 0,105 | 0,033 | **1,24×10^-05^** |
| *Terf1* | 0,070 | 0,054 | 0,686 | 0,00 | 0,00 | 1 | 0,061 | 0,068 | 0,951 | 0,044 | 0,039 | 0,892 |
| *Tgfb1* | 0,069 | 0,061 | 0,902 | 0,09 | 0,10 | 0,930 | 0,079 | 0,101 | 0,664 | 0,070 | 0,108 | 0,186 |
| *Tnf* | 0,001 | 0,019 | **0,003** | 0,00 | 0,04 | **3,97×10^-04^** | 0,002 | 0,032 | **3,81×10^-05^** | 0,002 | 0,023 | **2,71×10^-04^** |
| *Trp53* | 0,096 | 0,145 | 0,334 | 0,10 | 0,22 | **0,045** | 0,085 | 0,178 | **0,009** | 0,114 | 0,151 | 0,458 |

Table S3: AMs – strain effects; significant differences with p < 0,1 are bold; significant upregulation is black and downregulation gray

| **Gene** | ctrl | | | LPS | | | ctrl | | | LPS | | |
| --- | --- | --- | --- | --- | --- | --- | --- | --- | --- | --- | --- | --- |
|  | Sftpc-Ai9 | Sftpc-Ai9-Trf1 | p-Value | Sftpc-Ai9 | Sftpc-Ai9-Trf1 | p-Value | Lyz2-Ai9 | Lyz2-Ai9-Trf1 | p-Value | Lyz2-Ai9 | Lyz2-Ai9-Trf1 | p-Value |
|  | Average | Average |  | Average | Average |  | Average | Average |  | Average | Average |  |
| *Ccl3* | 0,062 | 0,052 | 1 | 1,680 | 2,944 | 0,839 | 0,065 | 0,056 | 1 | 3,106 | 3,061 | 1 |
| *Cdkn1a* | 0,664 | 0,636 | 1 | 1,163 | 1,285 | 1 | 0,765 | 3,762 | **1,33×10^-11^** | 1,971 | 4,466 | **0,082** |
| *Cdkn2b* | 0,008 | 0,006 | 1 | 0,025 | 0,021 | 1 | 0,010 | 0,036 | 1 | 0,071 | 0,034 | 1 |
| *Cxcl1* | 0,013 | 0,016 | 1 | 0,155 | 0,338 | 1 | 0,012 | 0,032 | 1 | 0,439 | 0,178 | 1 |
| *Cxcl5* | 0,011 | 0,016 | 1 | 0,008 | 0,005 | 1 | 0,029 | 0,052 | 1 | 0,042 | 0,057 | 1 |
| *Foxo3* | 0,086 | 0,105 | 1 | 0,112 | 0,073 | 1 | 0,087 | 0,147 | 1 | 0,131 | 0,120 | 1 |
| *Gadd45b* | 0,057 | 0,043 | 1 | 0,224 | 0,306 | 1 | 0,065 | 0,068 | 1 | 0,322 | 0,424 | 1 |
| *Gadd45g* | 0,450 | 0,537 | 1 | 0,407 | 0,241 | 1 | 0,666 | 0,441 | 1 | 0,184 | 0,327 | 1 |
| *H2afx* | 0,312 | 0,192 | 1 | 0,333 | 0,322 | 1 | 0,128 | 0,151 | 1 | 0,421 | 0,310 | 1 |
| *Hmox1* | 0,679 | 0,504 | 1 | 5,929 | 3,134 | 0,861 | 0,885 | 1,005 | 1 | 4,476 | 2,608 | 1 |
| *Hspa1a* | 0,036 | 0,047 | 1 | 0,005 | 0,003 | 1 | 0,039 | 0,119 | 1 | 0,011 | 0,017 | 1 |
| *Il18* | 2,389 | 2,589 | 1 | 2,522 | 2,153 | 1 | 2,861 | 2,805 | 1 | 2,264 | 2,889 | 1 |
| *Il1b* | 0,171 | 0,118 | 1 | 3,128 | 3,703 | 1 | 0,162 | 0,151 | 1 | 11,983 | 2,723 | **6,70×10^-06^** |
| *Il6* | 0,000 | 0,002 | 1 | 0,038 | 0,091 | 1 | 0,000 | 0,000 | 1 | 0,081 | 0,029 | 1 |
| *Lmnb1* | 0,140 | 0,101 | 1 | 0,137 | 0,158 | 1 | 0,051 | 0,036 | 1 | 0,152 | 0,075 | 1 |
| *Mtor* | 0,049 | 0,062 | 1 | 0,104 | 0,153 | 1 | 0,072 | 0,052 | 1 | 0,173 | 0,178 | 1 |
| *Nfe2l2* | 0,623 | 0,696 | 1 | 0,743 | 0,670 | 1 | 0,982 | 0,723 | 1 | 0,966 | 0,665 | 1 |
| *Rgcc* | 0,183 | 0,143 | 1 | 1,850 | 3,212 | 0,664 | 0,152 | 0,226 | 1 | 1,673 | 5,916 | **8,61×10^-06^** |
| *Saa3* | 0,276 | 0,279 | 1 | 2,392 | 7,548 | 0,157 | 0,478 | 0,485 | 1 | 4,415 | 5,761 | 1 |
| *Sirt1* | 0,062 | 0,068 | 1 | 0,097 | 0,060 | 1 | 0,070 | 0,072 | 1 | 0,085 | 0,075 | 1 |
| *Terf1* | 0,072 | 0,066 | 1 | 0,107 | 0,060 | 1 | 0,068 | 0,052 | 1 | 0,088 | 0,034 | 1 |
| *Tgfb1* | 0,807 | 0,833 | 1 | 0,901 | 0,888 | 1 | 1,170 | 1,045 | 1 | 1,780 | 1,278 | 1 |
| *Tnf* | 0,109 | 0,081 | 1 | 0,626 | 0,748 | 1 | 0,072 | 0,163 | 1 | 0,923 | 0,470 | 0,735 |
| *Trp53* | 0,292 | 0,289 | 1 | 0,550 | 0,553 | 1 | 0,263 | 0,238 | 1 | 0,640 | 0,516 | 1 |

Table S4: AMs – LPS effects; significant differences with p < 0,1 are bold; significant upregulation is black and downregulation gray

| **Gene** | Sftpc-Ai9 | | | Sftpc-Ai9-Trf1 | | | Lyz2-Ai9 | | | Lyz2-Ai9-Trf1 | | |
| --- | --- | --- | --- | --- | --- | --- | --- | --- | --- | --- | --- | --- |
|  | ctrl | LPS | p-Value | ctrl | LPS | p-Value | ctrl | LPS | p-Value | ctrl | LPS | p-Value |
|  | Average | Average |  | Average | Average |  | Average | Average |  | Average | Average |  |
| *Ccl3* | 0,068 | 0,935 | **2,18×10^-17^** | 0,066 | 1,886 | **8,18×10^-25^** | 0,077 | 1,735 | **4,80×10^-21^** | 0,066 | 1,727 | **3,19×10^-17^** |
| *Cdkn1a* | 0,726 | 0,647 | 0,839 | 0,800 | 0,823 | 1 | 0,905 | 1,101 | 0,728 | 4,495 | 2,519 | **0,020** |
| *Cdkn2b* | 0,009 | 0,014 | 0,791 | 0,007 | 0,013 | 0,868 | 0,011 | 0,040 | 0,266 | 0,043 | 0,019 | 0,551 |
| *Cxcl1* | 0,014 | 0,086 | **2,18×10^-04^** | 0,020 | 0,216 | **1,23×10^-05^** | 0,014 | 0,245 | **4,93×10^-05^** | 0,038 | 0,100 | 0,373 |
| *Cxcl5* | 0,012 | 0,004 | 0,549 | 0,020 | 0,003 | 0,386 | 0,034 | 0,024 | 0,763 | 0,062 | 0,032 | 0,556 |
| *Foxo3* | 0,095 | 0,062 | 0,425 | 0,132 | 0,047 | **0,005** | 0,103 | 0,073 | 0,712 | 0,176 | 0,068 | **0,041** |
| *Gadd45b* | 0,062 | 0,125 | 0,100 | 0,054 | 0,196 | **9,97×10^-05^** | 0,077 | 0,180 | **0,027** | 0,081 | 0,239 | **0,007** |
| *Gadd45g* | 0,492 | 0,227 | **0,002** | 0,676 | 0,155 | **4,40×10^-10^** | 0,788 | 0,103 | **4,88×10^-15^** | 0,527 | 0,184 | **3,02×10^-04^** |
| *H2afx* | 0,341 | 0,186 | **0,046** | 0,241 | 0,206 | 0,800 | 0,151 | 0,235 | 0,304 | 0,180 | 0,175 | 1 |
| *Hmox1* | 0,742 | 3,299 | **5,09×10^-07^** | 0,634 | 2,007 | **0,001** | 1,048 | 2,500 | **0,019** | 1,201 | 1,471 | 0,872 |
| *Hspa1a* | 0,039 | 0,003 | 0,213 | 0,059 | 0,002 | **2,32×10^-05^** | 0,046 | 0,006 | **0,032** | 0,142 | 0,010 | **0,009** |
| *Il18* | 2,613 | 1,403 | **0,005** | 3,259 | 1,379 | **2,40×10^-05^** | 3,386 | 1,265 | **1,17×10^-06^** | 3,351 | 1,630 | **9,03×10^-04^** |
| *Il1b* | 0,187 | 1,740 | **2,69×10^-15^** | 0,149 | 2,371 | **3,82×10^-22^** | 0,191 | 6,695 | **2,71×10^-34^** | 0,180 | 1,536 | **1,42×10^-09^** |
| *Il6* | 0,000 | 0,021 | **0,008** | 0,002 | 0,058 | **7,19×10^-05^** | 0,000 | 0,045 | **5,14×10^-04^** | 0,000 | 0,016 | 0,353 |
| *Lmnb1* | 0,153 | 0,076 | **0,060** | 0,127 | 0,101 | 0,777 | 0,060 | 0,085 | 0,667 | 0,043 | 0,042 | 1 |
| *Mtor* | 0,054 | 0,058 | 1 | 0,078 | 0,098 | 0,773 | 0,086 | 0,097 | 0,951 | 0,062 | 0,100 | 0,590 |
| *Nfe2l2* | 0,681 | 0,413 | **0,063** | 0,876 | 0,429 | **0,002** | 1,162 | 0,540 | **7,46×10^-04^** | 0,864 | 0,375 | **6,94×10^-04^** |
| *Rgcc* | 0,200 | 1,029 | **4,55×10^-12^** | 0,180 | 2,057 | **7,34×10^-22^** | 0,180 | 0,935 | **8,49×10^-10^** | 0,271 | 3,337 | **9,74×10^-20^** |
| *Saa3* | 0,301 | 1,331 | **2,02×10^-06^** | 0,351 | 4,834 | **1,34×10^-08^** | 0,565 | 2,467 | **8,48×10^-04^** | 0,579 | 3,249 | **4,01×10^-04^** |
| *Sirt1* | 0,068 | 0,054 | 0,775 | 0,085 | 0,038 | 0,104 | 0,083 | 0,047 | 0,503 | 0,085 | 0,042 | 0,365 |
| *Terf1* | 0,078 | 0,059 | 0,727 | 0,083 | 0,038 | 0,157 | 0,080 | 0,049 | 0,576 | 0,062 | 0,019 | 0,200 |
| *Tgfb1* | 0,883 | 0,501 | **0,023** | 1,049 | 0,569 | **0,009** | 1,385 | 0,994 | 0,281 | 1,248 | 0,721 | **0,039** |
| *Tnf* | 0,119 | 0,348 | **0,003** | 0,102 | 0,479 | **1,02×10^-07^** | 0,086 | 0,516 | **1,52×10^-09^** | 0,195 | 0,265 | 0,615 |
| *Trp53* | 0,319 | 0,306 | 1 | 0,363 | 0,354 | 1 | 0,311 | 0,358 | 0,827 | 0,285 | 0,291 | 1 |

Table S5: Mo/IMs – strain effects; significant differences with p < 0,1 are bold; significant upregulation is black and downregulation gray

| **Gene** | ctrl | | | LPS | | | ctrl | | | LPS | | |
| --- | --- | --- | --- | --- | --- | --- | --- | --- | --- | --- | --- | --- |
|  | Sftpc-Ai9 | Sftpc-Ai9-Trf1 | p-Value | Sftpc-Ai9 | Sftpc-Ai9-Trf1 | p-Value | Lyz2-Ai9 | Lyz2-Ai9-Trf1 | p-Value | Lyz2-Ai9 | Lyz2-Ai9-Trf1 | p-Value |
|  | Average | Average |  | Average | Average |  | Average | Average |  | Average | Average |  |
| *Ccl3* | 0,084 | 0,168 | 1 | 1,868 | 1,633 | 1 | 0,086 | 0,098 | 1 | 2,487 | 2,474 | 1 |
| *Cdkn1a* | 0,271 | 0,323 | 1 | 2,071 | 1,818 | 1 | 0,324 | 0,586 | 1 | 2,482 | 2,482 | 1 |
| *Cdkn2b* | 0,000 | 0,009 | 1 | 0,005 | 0,004 | 1 | 0,006 | 0,000 | 1 | 0,012 | 0,015 | 1 |
| *Cxcl1* | 0,071 | 0,004 | 1 | 0,099 | 0,144 | 1 | 0,024 | 0,023 | 1 | 0,096 | 0,176 | 1 |
| *Cxcl5* | 0,000 | 0,000 | 1 | 0,028 | 0,108 | 1 | 0,031 | 0,015 | 1 | 0,031 | 0,042 | 1 |
| *Foxo3* | 0,116 | 0,080 | 1 | 0,071 | 0,135 | 1 | 0,073 | 0,158 | 1 | 0,142 | 0,126 | 1 |
| *Gadd45b* | 0,032 | 0,049 | 1 | 0,504 | 0,454 | 1 | 0,134 | 0,105 | 1 | 0,555 | 0,593 | 1 |
| *Gadd45g* | 0,078 | 0,044 | 1 | 0,136 | 0,112 | 1 | 0,043 | 0,038 | 1 | 0,123 | 0,092 | 1 |
| *H2afx* | 0,058 | 0,049 | 1 | 0,202 | 0,108 | 1 | 0,061 | 0,150 | 1 | 0,341 | 0,195 | 1 |
| *Hmox1* | 0,388 | 0,460 | 1 | 8,852 | 14,740 | 1 | 0,391 | 0,421 | 1 | 6,015 | 12,307 | 0,737 |
| *Hspa1a* | 0,006 | 0,044 | 1 | 0,028 | 0,018 | 1 | 0,073 | 0,225 | 1 | 0,041 | 0,023 | 1 |
| *Il18* | 0,194 | 0,146 | 1 | 0,400 | 0,261 | 1 | 0,104 | 0,150 | 1 | 0,284 | 0,210 | 1 |
| *Il1b* | 0,336 | 0,053 | 1 | 5,995 | 5,098 | 1 | 1,021 | 0,090 | 1 | 7,634 | 6,230 | 1 |
| *Il6* | 0,000 | 0,000 | 1 | 0,104 | 0,076 | 1 | 0,000 | 0,000 | 1 | 0,072 | 0,099 | 1 |
| *Lmnb1* | 0,013 | 0,062 | 1 | 0,179 | 0,220 | 1 | 0,067 | 0,075 | 1 | 0,384 | 0,306 | 1 |
| *Mtor* | 0,045 | 0,031 | 1 | 0,165 | 0,189 | 1 | 0,043 | 0,038 | 1 | 0,161 | 0,218 | 1 |
| *Nfe2l2* | 0,594 | 0,535 | 1 | 1,139 | 0,895 | 1 | 0,581 | 0,676 | 1 | 1,216 | 0,880 | 1 |
| *Rgcc* | 0,071 | 0,022 | 1 | 0,174 | 0,315 | 1 | 0,067 | 0,053 | 1 | 0,223 | 0,245 | 1 |
| *Saa3* | 5,149 | 0,782 | 1 | 64,310 | 100,980 | 1 | 3,148 | 0,526 | 1 | 66,785 | 106,805 | 1 |
| *Sirt1* | 0,052 | 0,022 | 1 | 0,052 | 0,081 | 1 | 0,073 | 0,023 | 1 | 0,108 | 0,073 | 1 |
| *Terf1* | 0,039 | 0,062 | 1 | 0,071 | 0,148 | 1 | 0,122 | 0,053 | 1 | 0,118 | 0,065 | 1 |
| *Tgfb1* | 0,640 | 0,530 | 1 | 1,087 | 1,431 | 1 | 0,776 | 0,496 | 1 | 1,670 | 1,534 | 1 |
| *Tnf* | 0,103 | 0,075 | 1 | 0,593 | 0,490 | 1 | 0,043 | 0,038 | 1 | 0,738 | 0,459 | 1 |
| *Trp53* | 0,226 | 0,243 | 1 | 0,494 | 0,625 | 1 | 0,244 | 0,195 | 1 | 0,601 | 0,478 | 1 |

Table S6: Mo/IMs – LPS effects; significant differences with p < 0,1 are bold; significant upregulation is black and downregulation gray

| **Gene** | Sftpc-Ai9 | | | Sftpc-Ai9-Trf1 | | | Lyz2-Ai9 | | | Lyz2-Ai9-Trf1 | | |
| --- | --- | --- | --- | --- | --- | --- | --- | --- | --- | --- | --- | --- |
|  | ctrl | LPS | p-Value | ctrl | LPS | p-Value | ctrl | LPS | p-Value | ctrl | LPS | p-Value |
|  | Average | Average |  | Average | Average |  | Average | Average |  | Average | Average |  |
| *Ccl3* | 0,143 | 1,291 | **0,016** | 0,236 | 0,929 | **0,056** | 0,173 | 2,104 | 0,133 | 0,176 | 1,863 | 0,226 |
| *Cdkn1a* | 0,462 | 1,430 | **0,052** | 0,453 | 1,034 | **0,044** | 0,655 | 2,100 | 0,111 | 1,054 | 1,868 | 0,549 |
| *Cdkn2b* | 0,000 | 0,003 | 1 | 0,012 | 0,003 | 0,574 | 0,012 | 0,010 | 1 | 0,000 | 0,012 | 1 |
| *Cxcl1* | 0,121 | 0,068 | 0,679 | 0,006 | 0,082 | **0,077** | 0,049 | 0,081 | 1 | 0,041 | 0,132 | 0,877 |
| *Cxcl5* | 0,000 | 0,020 | 0,785 | 0,000 | 0,061 | 1 | 0,062 | 0,026 | 0,618 | 0,027 | 0,032 | 1 |
| *Foxo3* | 0,198 | 0,049 | **0,024** | 0,112 | 0,077 | 0,646 | 0,148 | 0,120 | 0,981 | 0,284 | 0,095 | **0,095** |
| *Gadd45b* | 0,055 | 0,348 | **0,010** | 0,068 | 0,259 | **0,007** | 0,272 | 0,470 | 0,762 | 0,189 | 0,446 | 0,445 |
| *Gadd45g* | 0,132 | 0,094 | 0,800 | 0,062 | 0,064 | 1 | 0,087 | 0,104 | 1 | 0,068 | 0,069 | 1 |
| *H2afx* | 0,099 | 0,140 | 0,936 | 0,068 | 0,061 | 0,997 | 0,124 | 0,289 | 0,541 | 0,270 | 0,147 | 0,488 |
| *Hmox1* | 0,660 | 6,115 | **0,003** | 0,645 | 8,388 | **2,21×10^-08^** | 0,791 | 5,088 | **0,046** | 0,757 | 9,264 | **5,36×10^-04^** |
| *Hspa1a* | 0,011 | 0,020 | 1 | 0,062 | 0,010 | **0,069** | 0,148 | 0,035 | 0,324 | 0,405 | 0,017 | **9,82×10^-06^** |
| *Il18* | 0,330 | 0,276 | 0,885 | 0,205 | 0,148 | 0,626 | 0,210 | 0,240 | 1 | 0,270 | 0,158 | 0,553 |
| *Il1b* | 0,572 | 4,142 | **0,033** | 0,074 | 2,901 | **7,18×10^-06^** | 2,064 | 6,457 | 0,471 | 0,162 | 4,690 | **0,011** |
| *Il6* | 0,000 | 0,072 | 0,875 | 0,000 | 0,044 | 0,174 | 0,000 | 0,061 | 0,731 | 0,000 | 0,075 | 0,569 |
| *Lmnb1* | 0,022 | 0,124 | 0,146 | 0,087 | 0,125 | 0,695 | 0,136 | 0,325 | 0,518 | 0,135 | 0,230 | 0,760 |
| *Mtor* | 0,077 | 0,114 | 0,903 | 0,043 | 0,108 | 0,257 | 0,087 | 0,136 | 0,970 | 0,068 | 0,164 | 0,557 |
| *Nfe2l2* | 1,013 | 0,787 | 0,682 | 0,751 | 0,510 | 0,320 | 1,174 | 1,028 | 0,986 | 1,216 | 0,662 | 0,217 |
| *Rgcc* | 0,121 | 0,120 | 1 | 0,031 | 0,179 | **0,002** | 0,136 | 0,189 | 1 | 0,095 | 0,184 | 0,923 |
| *Saa3* | 8,773 | 44,428 | **0,004** | 1,098 | 57,461 | **1,26×10^-20^** | 6,364 | 56,491 | **5,54×10^-04^** | 0,946 | 80,398 | **5,90×10^-14^** |
| *Sirt1* | 0,088 | 0,036 | 0,417 | 0,031 | 0,046 | 0,941 | 0,148 | 0,091 | 0,769 | 0,041 | 0,055 | 1 |
| *Terf1* | 0,066 | 0,049 | 0,877 | 0,087 | 0,084 | 1 | 0,247 | 0,100 | 0,261 | 0,095 | 0,049 | 0,647 |
| *Tgfb1* | 1,090 | 0,751 | 0,490 | 0,744 | 0,814 | 1 | 1,569 | 1,413 | 1 | 0,892 | 1,154 | 0,899 |
| *Tnf* | 0,176 | 0,410 | 0,452 | 0,105 | 0,279 | **0,076** | 0,087 | 0,624 | 1 | 0,068 | 0,345 | 0,162 |
| *Trp53* | 0,385 | 0,341 | 0,967 | 0,341 | 0,356 | 1 | 0,494 | 0,508 | 1 | 0,351 | 0,360 | 1 |
