# Supplementary material for: Telomere damage-mediated senescence in alveolar epithelial type II cells but not in macrophages aggravates inflammation in acute lung injury
Source: Respir Res. 2026 Mar 14;27:161. doi: 10.1186/s12931-026-03627-0 (PMC13063740; doi:10.1186/s12931-026-03627-0)

Telomere damage-mediated senescence in alveolar epithelial type II cells but not in macrophages aggravates inflammation in acute lung injury

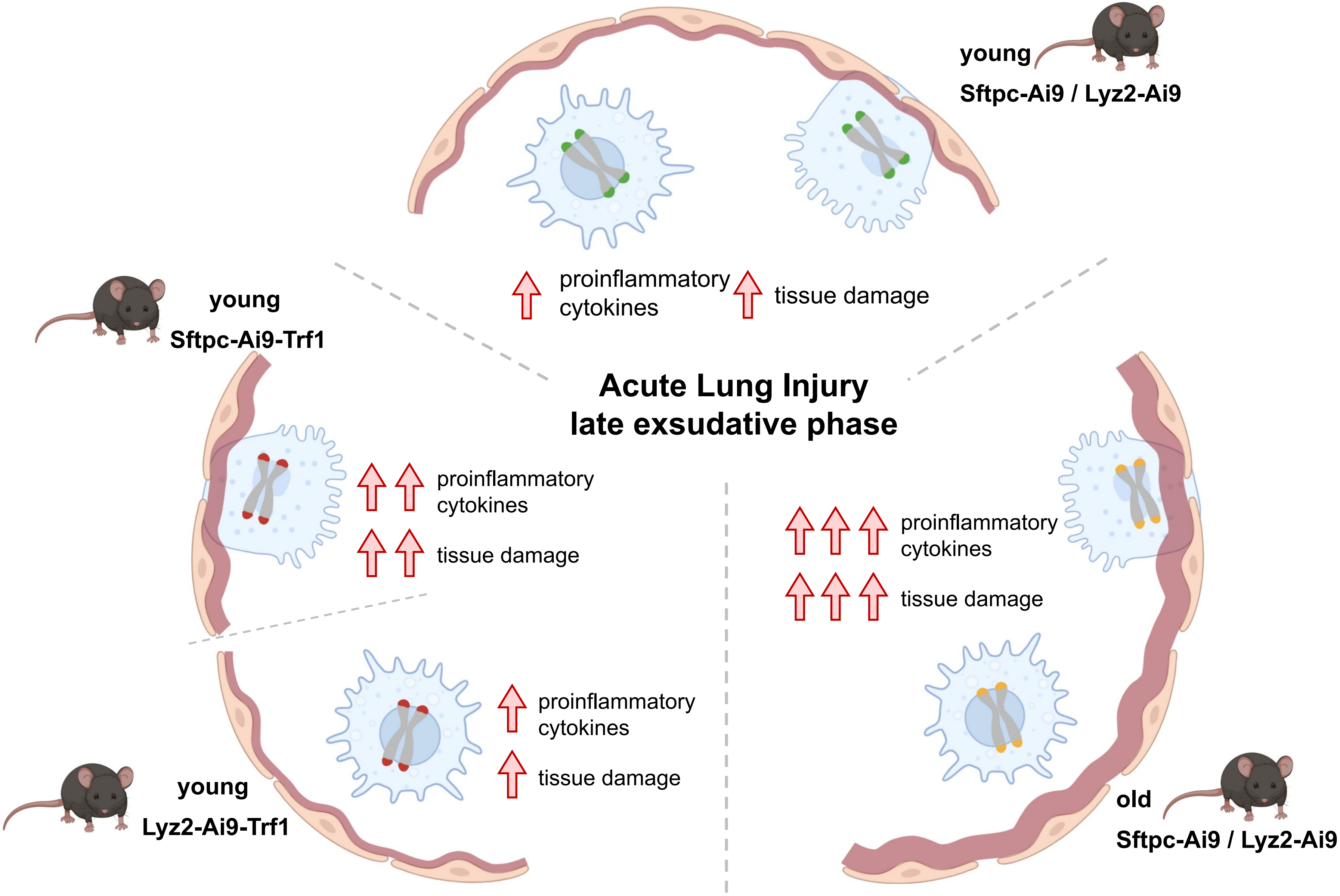

Supplement: Supplementary file 3 — Supplementary Material 3. [file 12931_2026_3627_MOESM3_ESM.pdf]
